# Transcriptomic Analysis of CRISPR/Cas9-Mediated PARP1-Knockout Cells under the Influence of Topotecan and TDP1 Inhibitor

**DOI:** 10.3390/ijms24065148

**Published:** 2023-03-07

**Authors:** Nadezhda S. Dyrkheeva, Anastasia A. Malakhova, Aleksandra L. Zakharenko, Larisa S. Okorokova, Dmitriy N. Shtokalo, Sophia V. Pavlova, Sergey P. Medvedev, Suren M. Zakian, Anna A. Nushtaeva, Alexey E. Tupikin, Marsel R. Kabilov, Svetlana N. Khodyreva, Olga A. Luzina, Nariman F. Salakhutdinov, Olga I. Lavrik

**Affiliations:** 1 Institute of Chemical Biology and Fundamental Medicine, Siberian Branch of the Russian Academy of Sciences, 8 Lavrentyeva Ave., 630090 Novosibirsk, Russia; 2 Federal Research Centre Institute of Cytology and Genetics, Siberian Branch of the Russian Academy of Sciences, 10 Lavrentyeva Ave., 630090 Novosibirsk, Russia; 3 AcademGene LLC, 6 Lavrentyeva Ave., 630090 Novosibirsk, Russia; 4 A.P. Ershov Institute of Informatics Systems SB RAS, 6 Lavrentyeva Ave., 630090 Novosibirsk, Russia; 5 N.N. Vorozhtsov Novosibirsk Institute of Organic Chemistry, Siberian Branch of the Russian Academy of Sciences, 9 Lavrentyeva Ave., 630090 Novosibirsk, Russia; 6 Department of Molecular Biology and Biotechnology, Novosibirsk State University, 630090 Novosibirsk, Russia

**Keywords:** poly(ADP-ribose) polymerase 1, HEK293A, transcriptome, PARP1 knockout, topoisomerase 1, tyrosyl-DNA phosphodiesterase 1, TDP1 inhibitor, topotecan, DMSO, DNA repair

## Abstract

Topoisomerase 1 (TOP1) is an enzyme that regulates DNA topology and is essential for replication, recombination, and other processes. The normal TOP1 catalytic cycle involves the formation of a short-lived covalent complex with the 3′ end of DNA (TOP1 cleavage complex, TOP1cc), which can be stabilized, resulting in cell death. This fact substantiates the effectiveness of anticancer drugs—TOP1 poisons, such as topotecan, that block the relegation of DNA and fix TOP1cc. Tyrosyl-DNA phosphodiesterase 1 (TDP1) is able to eliminate TOP1cc. Thus, TDP1 interferes with the action of topotecan. Poly(ADP-ribose) polymerase 1 (PARP1) is a key regulator of many processes in the cell, such as maintaining the integrity of the genome, regulation of the cell cycle, cell death, and others. PARP1 also controls the repair of TOP1cc. We performed a transcriptomic analysis of wild type and PARP1 knockout HEK293A cells treated with topotecan and TDP1 inhibitor OL9-119 alone and in combination. The largest number of differentially expressed genes (DEGs, about 4000 both up- and down-regulated genes) was found in knockout cells. Topotecan and OL9-119 treatment elicited significantly fewer DEGs in WT cells and negligible DEGs in PARP1-KO cells. A significant part of the changes caused by PARP1-KO affected the synthesis and processing of proteins. Differences under the action of treatment with TOP1 or TDP1 inhibitors alone were found in the signaling pathways for the development of cancer, DNA repair, and the proteasome. The drug combination resulted in DEGs in the ribosome, proteasome, spliceosome, and oxidative phosphorylation pathways.

## 1. Introduction

The synthesis of poly(ADP-ribose) (PAR) is an immediate response of cells to DNA damage catalyzed by poly(ADP-ribose) polymerases, which transfer ADP-ribose units from NAD^+^ onto target molecules [1]. PAR is a linear and branched polymer up to 200 units long that is covalently attached to the target proteins, including PARP itself, as well as to DNA and RNA [2,3]. PARP1 is the most abundant member of the PARP family, presenting more in the cell nucleus and less in the cytosol [4]. PARP1 is considered primarily as a regulator of DNA base excision repair, including the repair of single-strand breaks. PARP1 binds to damaged DNA and forms DNA repair foci, coordinating the repair process, although its involvement in the regulation of other DNA repair processes is well documented [2,5,6,7]. The PAR polymer performs various signaling functions, both being covalently attached to targets and in the free state. In the cell, there is a dynamic balance between the synthesis of PAR and its degradation by the corresponding enzymes. Beyond DNA repair, PAR is involved in the regulation of the chromatin structure, cell cycle control, cell death, and mRNA stability [2,3,8,9].

DNA topoisomerase 1 (TOP1) is an enzyme that controls and alters the topologic states of DNA, catalyzing the transient breaking and rejoining of DNA. TOP1 wraps around double-stranded DNA and cuts one of the two strands, creating a 3’ phosphotyrosyl DNA-TOP1 intermediate [10]. The 5’ end is then free to rotate around the intact strand, relaxing the supercoil until TOP1 relegates the broken strand. These intermediates are normally short-lived, but in some cases they can be trapped, leading to the formation of the stable trapped DNA-TOP1 cleavage complex (TOP1cc) and the clamping of single-strand breaks. The “trap” might be TOP1 poisons such as topotecan (Tpc), which forms the ternary complex DNA-TOP1-Tpc and prevents the relegation of DNA. Tpc is a water soluble analog of the natural compound camptothecin (CPT), and is approved for the treatment of small cell lung carcinoma and cervical and ovarian cancer [11]. Thus, TOP1 trapping on the DNA by specific TOP1 poisons triggers double-strand DNA breaks and cell death. A number of observations support the involvement of PARP1 in the repair of TOP1cc. PARP1-deficient cells are hypersensitive to CPT [12,13,14]. PAR accumulates in cells after CPT-treatment [15,16,17,18,19]. Furthermore, in the presence of PARP inhibitors, TOP1cc repair is suppressed and sensitivity to CPT and its derivatives is increased [14,15,16,17,20]. Moreover, PARP1 inhibiting hinders the release of TOP1 from TOP1cc [21,22,23,24].

Tyrosyl-DNA phosphodiesterase 1 (TDP1) is a key DNA repair enzyme for trapped TOP1cc. The main function of TDP1 is the hydrolysis of the phosphodiester bond between the DNA 3’ end and the TOP1 tyrosyl moiety [25]. TDP1 is also able to repair the 3’ ends of DNA from covalent adducts of various origins [26], and has other activities [27,28]. TDP1 prevents the action of Tpc, and it is one of the factors of resistance of tumor cells to this drug [29,30,31]. TDP1 is overexpressed in various types of cancer [29,32,33]. TDP1 deficiency increases the likelihood of TOP1-SSB and DSB formation [34,35]. The inhibition of TDP1 sensitizes tumor cells to TOP1 poisons [36,37,38,39,40,41,42,43,44,45,46,47,48,49,50,51,52,53,54,55,56]. Thus, TDP1 plays an important role in maintaining genome stability and is considered as a potential therapeutic target in cancer treatment [11]. Our team discovered a number of TDP1 inhibitors among derivatives of natural compounds [11,57], including usnic acid (UA) derivatives [40,48,49,52,55]. The compound OL9-119 is a UA derivative that efficiently inhibits TDP1 and has a pronounced sensitizing effect on the Tpc cytotoxicity in vitro [55, compound 20d] and on the antitumor effect of Tpc in vivo [55,58]. The N-terminal domain of TDP1 directly binds the C-terminal domain of PARP1, and TDP1 is PARylated by PARP1 in order to be recruited to TOP1cc without inactivation. The PARylation recruits TDP1 to DNA damage sites. PARylation stabilizes TDP1, increasing the half-life of TDP1 [20].

The elimination of TOP1cc by TDP1 requires the preliminary proteolytic degradation of the TOP1 protein globule, since TDP1 cannot hydrolyze covalent phosphotyrosyl bonds if the TOP1cc is in the native conformation [25]. This degradation is a complex multi-step process, and can proceed via several mechanisms [59]. The primary one is the cleavage by the 26S proteasome. The PARylation of the TOP1cc controls this process, since sustained PARylation blocked the repair of TOP1cc [60]. PARylation of TOP1cc is transient and serves as a signaling mechanism for TDP1 recruitment to the site of damage [18,20,61,62]. The temporary nature of the modification is due to the fact that PAR molecules activate deubiquitination and block the proteolysis of TOP1cc [60]. PAR molecules remain bound to TOP1cc until TDP1 is recruited, thereby protecting the complex from premature proteolysis. After complete TDP1 recruitment, the process of de-PARylation is activated, in which PARG plays the main role, removing PAR molecules [60]. Thus, the process of partial proteasomal degradation of the TOP1cc is activated, after which this complex is hydrolyzed by TDP1 with the formation of a single-strand break [59,60]. Another proteolytic pathway to eliminate TOP1cc is associated with the Spartan (SPRTN) protease. SPRTN is a DNA-binding metalloprotease which acts as an independent proteolytic mechanism to resolve TOP1cc as well as other types of DNA-protein crosslinks (DPC) in mammalian cells [63]. Recent studies have shown that PARylation of the TOP1cc is required not only for TDP1 recruitment but also for recruitment of the SPRTN metalloprotease [64]. A PAR-binding domain has been found in SPRTN which facilitates the binding of a metalloprotease to PAR chains synthesized by PARP1. In cells with mutant SPRTN that are unable to bind to PAR molecules, various DPC, including TOP1cc, accumulated [64]. Cells of the model organism *Caenorhabditis elegans* with mutant SPRTN are sensitive to drugs that induce DPC. Thus, SPRTN binding to PAR molecules is required for the repair of DPC. PARylation is the biochemical signal that marks DPC, including TOP1cc, for SPRTN-dependent proteolysis [64].

PARP1 is a nick sensor for trapped TOP1cc. It has been shown that PARP1 regulates the subnuclear dynamics of TOP1, delocalizing TOP1 from the nucleolus to the nucleoplasm. TOP1 is PARylated by PARP1, which serves to engage TOP1 to the active sites of nucleic acid synthesis [20]. Several authors discuss the rationale behind the use of the combination of TOP1 poison and TDP1 and PARP inhibitors in cancer treatment [20,24,35,65,66,67,68,69]. Thus, PARP1 was found to be critical in the repair of TOP1cc by TDP1.

**Figure 1:**
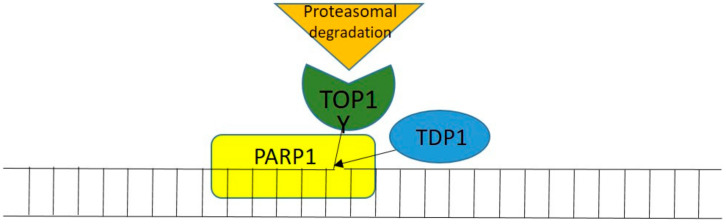
TOP1cc repair scheme due to TDP1 activity. PARP1 coordinates the process of TOP1 protein globule proteolysis to provide TDP1 access to the TOP1-DNA cross-linking site. PARP1 recruits TDP1 to the site of DNA damage via PARylation. TDP1 removes the blocking TOP1 oligopeptide at the 3′ end of the break, allowing DNA integrity to be restored.

DNA repair is important for cell survival following exposure to TOP1 poison. Under exposure to CPT-derived agents, tumor cells activate DNA repair. The interplay between DNA repair enzymes, namely PARP1 and TDP1, (Figure 1) allows cells to effectively remove DNA damage. The expression level or mutagenesis of the appropriate repair enzymes may be significant in the cell’s capacity to repair Tpc-induced DNA damage. In this work, we performed the transcriptome sequencing of HEK293A wild-type and PARP1-knockout (PARP1-KO) cells under the action of the TOP1 poison Tpc and the compound OL9-119, an effective TDP1 inhibitor. If TOP1cc damage is not repaired, the single-stranded DNA then breaks upon collision with the replication fork, and turns into double-strand breaks and leads to cell death. Such “trapped” TOP1 is degraded by proteases to a short oligopeptide, and then TDP1 removes the adduct [59]. Thus, exposing cells to the action of Tpc, we introduce damage to the DNA, and by adding OL9-119, we do not allow the DNA chain to be restored. Since PARP1 was found to be a key player in the repair of trapped TOP1cc by TDP1, we investigated the influence of these compounds separately and in combination with the gene expression of the wild type and PARP1-knockout (PARP1-KO) cells.

## 2. Results

We recently described the generation of an HEK293A cell line with the homozygous knockout of the *PARP1* gene using the CRISPR/Cas9 approach [38]. We determined by MTT test that the obtained PARP1-knockout (PARP1-KO) HEK293A cells were more sensitive to Tpc than wild-type (WT) HEK293A cells (50% cytotoxic concentration of sample on cells, CC_50_ 27 ± 4 nM and 50 ± 5 nM, respectively) [38]. We have previously found the effective TDP1 inhibitor compound OL9-119 (20d in publication [55]) with an IC_50_ of 26 nM (IC_50_ is a 50% inhibition concentration of sample dose in the experiments with purified recombinant TDP1). The OL9-119 compound showed a sensitizing effect on Tpc in vitro and in vivo. Here, we studied the effect of co-treatment with Tpc and OL9-119 in various combinations on the level of gene expression in WT and PARP1-KO HEK293A cells. cDNA libraries were created for all of the RNA samples, and the sequencing of the differentially expressed genes (DEGs) was then determined.

### 2.1. Samples Preparation

**Figure 2:**
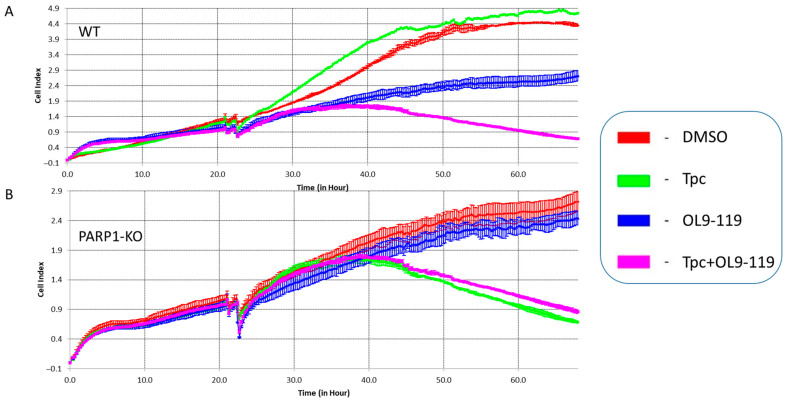
The influence of Topotecan and OL9-119 on cell viability according to an impedance-based real-time assay on xCelligence RTCA (Agilent). Cell growth of WT HEK293A (**A**) or PARP1-KO HEK293A (**B**) cells in the control wells without compounds added (red), with 25 nM Tpc (green), 5 µM OL9-119 (blue), or combination of Tpc and OL9-119 (magenta). The tested compounds were added to the medium 21 h after start.

A preliminary experiment on the selection of processing conditions was carried out to determine the dose and duration of chemical exposure. We studied the effect of Tpc and OL9-119 on the viability of WT and PARP1-KO HEK293A cells using the xCelligence instrument (Agilent, Santa Clara, CA, USA). The principle of the device operation is the real time detection of the impedance of the cell layer growing in the well of the plate. The resulting dependence curve indicates changes of the cell index (a parameter that reflects cell viability, adherence and proliferation) on time. The tested compounds were added to the medium after the formation of a ~50% cell layer (nearly 21 h after start, Figure 2A,B), and the cell culture was monitored for 50 h more. The addition of Tpc reduced cell viability, as it was found in our previous work by MTT test [38] that PARP1-KO HEK293A cells were more sensitive to Tpc than are WT HEK293A cells. Tpc in 25 nM concentration was cytotoxic for PARP1-KO, but not for WT cells (Figure 2). This Tpc concentration was equal to CC_50_ for PARP1-KO (27 ± 4 nM), and twice lower than CC_50_ for WT cells (50 ± 5 nM). We previously checked the cytotoxicity of the TDP1 inhibitor OL9-119 by MTT test (CC_50_ > 5 μM for WT and PARP1-KO cells). Using the xCelligence analysis, we found that 2 µM OL9-119 had a slight cytotoxic effect on WT HEK293A cells, and was not cytotoxic for PARP1-KO cells (Figure 2).

Based on the results obtained using the MTT test and the xCelligence instrument, processing conditions for the cell lines were selected for the RNA-seq experiment. The concentration conditions for chemical agents were chosen, as well as the time frame of the experiment: after plating 1 million cells per well in a six-well plate, the plates were kept in a CO_2_ incubator for 20–21 h to double the number of cells in the well. Since OL9-119 is not soluble in water, we used DMSO to dissolve the substance, and treated cells with 0.1% DMSO as a negative solvent control. DMSO was not cytotoxic in 0.1% concentration for both WT and PARP1-KO cells (Figure 2, red). We used a Tpc concentration twice higher than CC_50_ (100 nM for WT HEK293A or 50 nM for PARP1-KO cells). The RNA samples for the gene expression studies were prepared for WT and PARP1-KO HEK293A cell lines with different treatments:

1. No treatment,
2. DMSO 0.1%,
3. Tpc (100 nM for WT HEK293A or 50 nM for PARP1-KO cells) + DMSO 0.1%,
4. OL9-119 (5 μM),
5. Tpc + OL9-119 (100 nM for WT HEK293A or 50 nM for PARP1-KO cells, and 5 μM, respectively).

The appropriate reagents were added to the sample and left for five hours before TRIzol (TRI Reagent) treatment and RNA isolation.

### 2.2. PARP1 Knockout Effect

**Figure 3:**
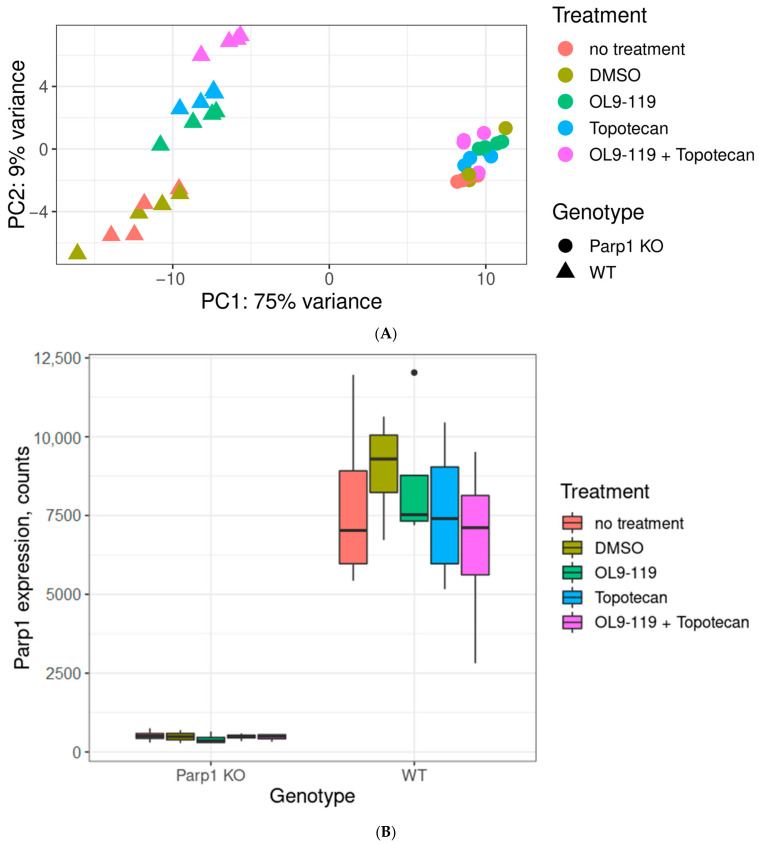
(**A**) Principal component analysis (PCA) plot illustrates the transcriptional pattern of cells by the profiles of gene expression in wild type (WT, triangles) and PARP1 knockout (PARP1-KO, circles) cells. Principal component analysis (PCA) plot illustrates the transcriptional pattern of cells by the profiles of gene expression in wild type (WT, triangles) and PARP1 knockout (PARP1-KO, circles) cells. Samples clustering based on a PCA of differentially expressed genes. The axes: the principal components PC1 and PC2 with the proportion of explained variance of the data for each principal component. (**B**) *PARP1* expression level in wild type (WT) and knockout (PARP1-KO) cells under treatment determined by RNAseq.

First, we analyzed the PARP1 knockout effect. A principal component analysis (PCA) reveals the clusterization of samples by the gene expression patterns. A high percentage of variability (84%) on the PCA plot was explained by the first two components. The change in the genotype of cells strongly affected the transcriptional pattern (Figure 3A). Thus, the greatest contribution to the difference between the samples was made by the *PARP1* knockout. We registered that WT HEK293A cells responded differently to treatment. The samples were divided into groups according to the type of the treatment on the PCA plot. All samples of PARP1-KO HEK293A cells with or without treatment demonstrated no PARP1 expression, which confirmed the successful knockout of the gene (Figure 3B).

**Figure 4:**
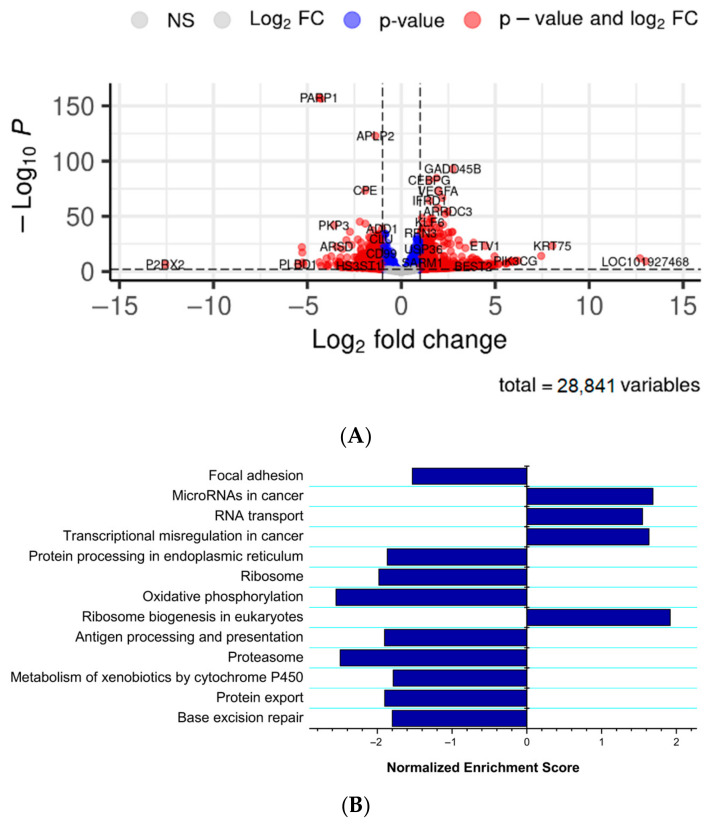
PARP1 knockout effect. (**A**) PARP1-KO cells versus WT HEK293A cells in DMSO 0.1%. Volcano Plot for differential gene expression (DEG); (**B**) Gene ontology (GO) enrichment analysis of DEGs when comparing HEK293A PARP1-KO cells with WT cells. Normalized enrichment scores indicate the distribution of Gene Ontology categories across a list of genes ranked by hypergeometrical score (HGS). Higher enrichment scores indicate a shift of genes belonging to certain GO categories towards either end of the ranked list, representing up or down regulation (positive or negative values, respectively).

When comparing WT cells with PARP1-KO cells > 4000 differentially expressed genes (DEGs) were found in PARP1-KO cells (2253 upregulated and 2079 downregulated genes). The transcriptional pattern has changed dramatically in PARP1-KO HEK293A cells versus WT cells (Figure 4).

By Gene Set Enrichment Analysis (GSEA), we revealed that DEGs of PARP1-KO versus WT cells (Figure 4B) were present in the processes associated with DNA base excision repair, oxidative phosphorylation, proteasome, ribosome, protein processing in endoplasmic reticulum, ribosome biogenesis in eukaryotes, microRNAs in cancer, antigen processing and presentation, transcriptional misregulation in cancer, focal adhesion, protein export, RNA transport, metabolism of xenobiotics by cytochrome P450, and other processes (Table S1).

It is known that the effects of DMSO should be considered, and DMSO is not inert, even at low concentrations [70]. DMSO is indispensable within the experiments because the used TDP1 inhibitor (OL9-119) is soluble in DMSO, thus before investigating the effect of this compound, it was necessary to find out how the solvent affects the transcriptome. We exposed WT and PARP1-KO HEK293A cells with or without 0.1% DMSO and analyzed the transcriptome profiles. DMSO was present in four out of five samples (2–5): (1) No treatment; (2) DMSO; (3) Tpc + DMSO; (4) OL9-119; (5) Tpc + OL9-119. We did not find a critical effect of 0.1% DMSO on the transcription pattern (Figure S2). Under the treatment, WT cells samples variability was significantly changed (Figure 3A), but the compounds (Tpc, OL9-119) practically did not act on PARP1-KO cells (Figure 3A). The genotype of the cells contributes the most to the difference between the samples. DEGs of the PARP1-KO versus WT cells (Figure 4B) both without DMSO and in the presence of DMSO were present in the same processes.

### 2.3. Topotecan Effects on Transcriptome

**Figure 5:**
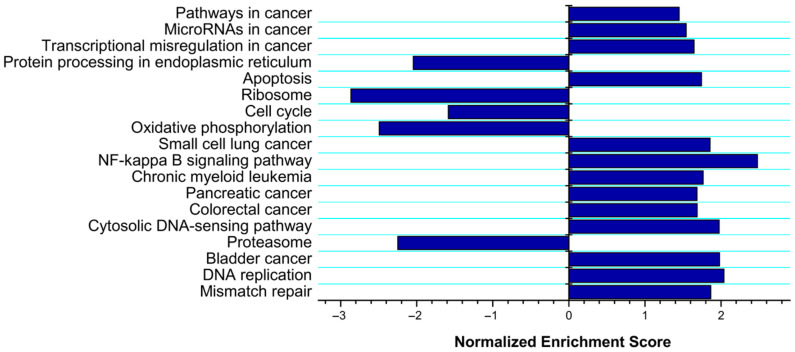
Topotecan effect on WT HEK293A cells treated with 100 nM Tpc vs. untreated cells. Gene Ontology (GO) enrichment analysis of DEGs when comparing HEK293A WT cells under Tpc treatment with HEK293A WT cells without treatment. Normalized enrichment scores indicate the distribution of Gene Ontology categories across a list of genes ranked by hypergeometrical score (HGS).

When treated with the anticancer drug Tpc, the greatest changes in gene expression were observed in WT cells (Figure S3), and the smallest were in PARP1-KO cells (Figure S3). When treated with Tpc, the highest degree of DEG was observed (Figure 5) in the signaling pathways associated with oxidative phosphorylation, the ribosome, protein processing in endoplasmic reticulum, proteasome, DNA replication, apoptosis, the NF-kappa B signaling pathway, the cytosolic DNA-sensing pathway, mismatch repair, cell cycle, pathways in cancer, transcriptional misregulation in cancer, microRNAs in cancer, different types of cancer (small cell lung cancer, bladder cancer, chronic myeloid leukemia, pancreatic cancer, colorectal cancer) and other processes (Table S2). In the PARP1-KO cell line, none of these pathways were identified. It was previously reported [71] that the treatment of human breast MCF-7 tumor cells with Tpc results in DEGs that regulate p53-dependent pathways as well as genes that regulate apoptosis. They also indicated the central role of ERα in Tpc cytotoxicity. Here, we can see also that Tpc WT HEK293A cells changes the expression level of protein genes involved in apoptosis, DNA repair, and cell cycle control.

### 2.4. Influence of TDP1 Inhibitor OL9-119 on Gene Expression Level

**Figure 6:**
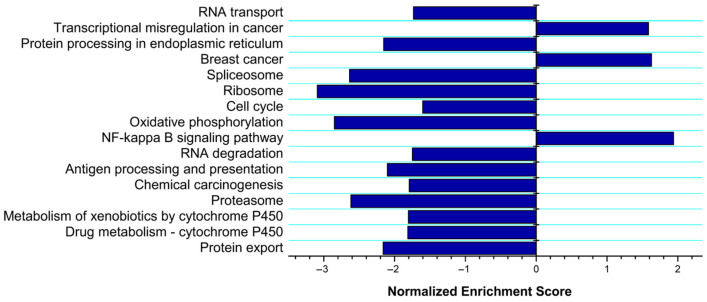
OL9-119’s effect on WT HEK293A cells treated with 5 µM OL9-119 vs. untreated cells. Gene Ontology (GO) enrichment analysis of DEGs when comparing HEK293A WT cells under OL9-119 treatment with HEK293A WT cells without treatment. Normalized enrichment scores indicate the distribution of Gene Ontology categories across a list of genes ranked by hypergeometrical score (HGS).

When treating WT HEK293A cells with the OL9-119 compound, they also were more susceptible to changes in the expression of proteins involved in different processes then PARP1-KO cells (Figure S4). DEGs (Figure 6) were present in processes associated with oxidative phosphorylation, the spliceosome, the proteasome, the ribosome, protein processing in the endoplasmic reticulum, antigen processing and presentation, protein export, the NF-kappa B signaling pathway, RNA transport, metabolism of xenobiotics by cytochrome P450, breast cancer, the cell cycle, transcriptional misregulation in cancer, RNA degradation, chemical carcinogenesis, drug metabolism by cytochrome P450, and other processes (Table S3). In the PARP1-KO cell line, none of these pathways was identified.

### 2.5. Co-Presence Effects of Topotecan and OL9-119

**Figure 7:**
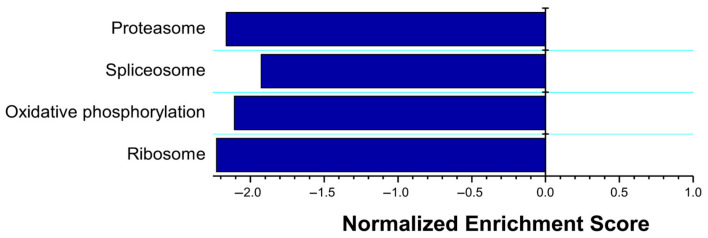
Effect of topotecan and OL9-119 combination on WT HEK293A cells treated with Tpc + OL9-119 vs. untreated cells. Gene Ontology (GO) enrichment analysis of DEGs when comparing HEK293A WT cells under Tpc + OL9-119 treatment with HEK293A WT cells without treatment. Normalized enrichment scores indicate the distribution of Gene Ontology categories across a list of genes ranked by hypergeometrical score (HGS).

When treated with both Tpc and OL9-119, the greatest changes in gene expression were also observed in WT cells, but not in PARP1-KO cells (Figure S5). When treating WT HEK293A cells with Tpc + OL9-119, the highest degree of DEG compared to only Tpc was observed (Table S4) in the signaling pathways associated with oxidative phosphorylation, the spliceosome, the proteasome, and the ribosome (Figure 7). In the PARP1-KO cell line under Tpc + OL9-119 treatment, none of these pathways was identified.

## 3. Discussion

Cancer is one of the leading causes of death worldwide. In recent decades, research has been aimed at finding drugs for targeted cancer therapy as an effective method of suppressing the growth of cancer cells [72,73,74]. Different types of cancers often have changes in the expression levels of one or more DNA repair genes, and both decreased and increased gene expression occurs due to mutations and epigenetic changes [75,76,77]. Chemotherapy and radiotherapy are partially based on the increased susceptibility of rapidly dividing cancer cells to DNA damage. The therapy could activate DNA repair systems, thus the inhibition of DNA repair enzymes is a promising therapeutic strategy for the treatment of oncological diseases.

PARP1 is the highest expressed member of the PARP family, being a major member in DNA base excision repair (BER) through its association with DNA single-strand breaks (SSB) and recruiting other repair proteins to the damage sites [78]. In addition, PARP1 participates in many cellular processes, including other DNA repair processes, recombination, cell proliferation, and death, as well as maintaining genomic stability [78]. PARP1 knockout mice are viable, fertile and do not develop early onset tumors. Cells isolated from these mice show an increased level of homologous recombination [79,80]. PARP1 knockout mice are sensitive to ionizing radiation, and the inhibition of PARP1 brings the modest sensitization of cells in culture to radiation. In certain cell lines, the chemical inhibition of PARP activity is also associated with a marked sensitization to very low doses of radiation [81].

It is known that PARP1 inhibition and mutations of PARP1 can disrupt the stability of the genome. It was demonstrated on various tumor models [82] that the inhibition of PARP makes mice susceptible to various carcinogenic agents. However, *PARP1*(-/-) mice were not prone to tumor formation, and transcriptomic analysis showed that the mice were protected from colitis. This protection had been associated with wide transcriptome changes in the colon [83]. Several PARP1 inhibitors were approved by FDA for the cancer treatment, olaparib being the first one in 2014 [84]. PARP1 inhibition was found to be effective in the treatment of tumors carrying mutations in *BRCA1/2* genes exploiting the concept of synthetic lethality [85]. PARP1 is mostly over-expressed in cancers [86], but in the work [87] low *PARP1* expression level was found in the clinical samples from the patients with gastric cancer. The inhibition of PARP1 activity could sensitize tumor cells to the action of chemotherapeutic drugs. It has been found that PARP1 acts as a nick sensor for trapped TOP1cc in the repair of TOP1cc by TDP1 with both TOP1 and TDP1 being PARylated [20]. The possible use of the combination of TOP1 poison and the PARP inhibitor in cancer treatment is widely discussed [20,24,65,67].

In this work, we checked the influence of TOP1 poison Tpc and TDP1 inhibitor OL9-119 on the transcriptome of HEK293A wild-type and PARP1-knockout cells. First, we found more than 4000 differentially expressed genes (DEG) in PARP1-KO cells compared to wild type cells (2253 up-regulated genes and 2079 down-regulated genes). The expression of genes involved in various pathways has changed in PARP1-KO cells, including DNA BER, transcriptional misregulation in cancer, the proteasome, and other processes (Figure 4).

**Figure 8:**
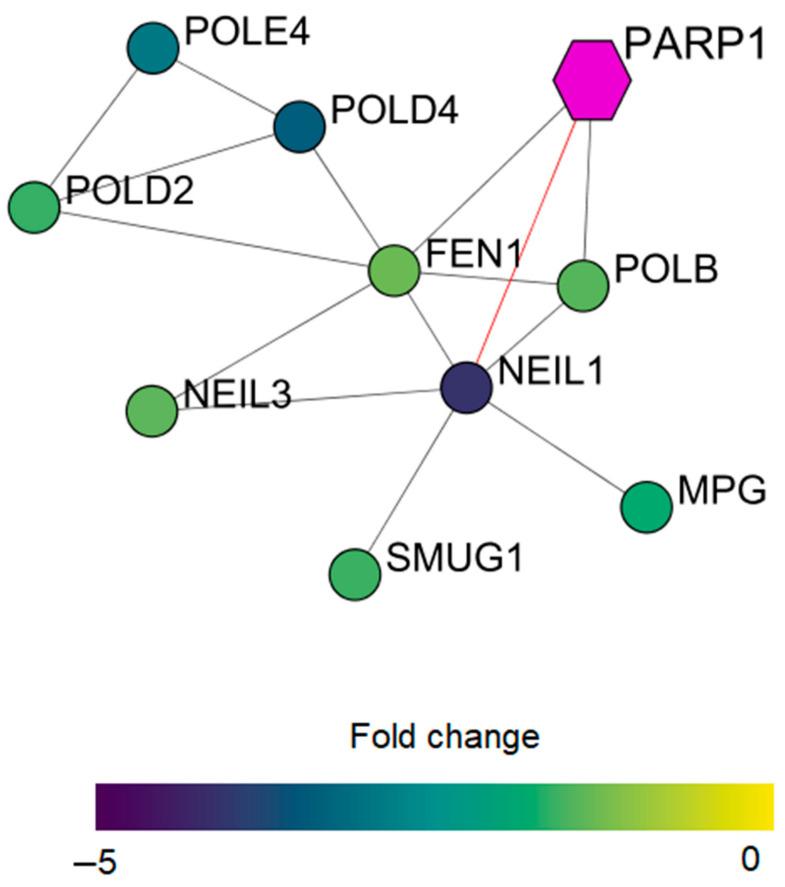
Network of protein-protein interactions of PARP1-dependent genes (green circles) obtained using the STRING tool. Pink hexagon—PARP1. The association between PARP1 and NEIL1 is highlighted in red [89]. The color intensity of nodes depends on the fold change in expression in PARP1 knockout cells compared to wild type cells. Rendered with Cytoscape v3.7.2. The PPI network was built with a minimum required interaction score of 0.7.

We found that PARP1 knockout causes dramatic changes in the BER genes’ transcription (Table 1, Figure 8). The expression level of DNA glycosylases genes (*NEIL1, SMUG1, MPG, NEIL3*), and DNA polymerases or their subunits genes (*POLE4, POLD2, POLD4, POLB*) decreased. The changes of expression levels of *POLE4, POLD4*, and *NEIL1* (Nei Like DNA Glycosylase 1) were most significant. NEIL1 and FEN1 (Flap Endonuclease 1) are the “junction” of the listed interactions (Figure 8). NEIL1 glycosylase initiates BER by removing damaged nitrogenous bases, mainly oxidized pyrimidines [88]. It was reported previously that the direct interaction of NEIL1 and PARP1 BRCT domain leads to the decrease of NEIL1 activity independent of PARylation [89]. PARP1 binds to the C-terminal domain of NEIL1 [89], as well as to other BER proteins [90,91,92]. In this work, we also demonstrated that NEIL1 is a “junction” for BER proteins. FEN1 removes 5’ overhanging “flaps” in DNA repair and processes the 5′ ends of Okazaki fragments in replication. DNA polymerases δ and ε are replicative polymerases [93] that are responsible for the synthesis of the lagging (DNA polymerase δ), or the leading (DNA polymerase ε) strand. These polymerases participate in the long-patch BER [94,95], but in the case of blocking damage they are usually replaced by repair DNA polymerases [96]. FEN1 inhibits strand displacement DNA synthesis catalyzed by DNA polymerase δ, but stimulates strand displacement synthesis catalyzed by BER DNA polymerase β, with direct DNA polymerase β-FEN1 interaction [97]. In PARP1-deficient cell extracts with reduced cellular expression of FEN1 and DNA ligase I, less efficient DNA repair was observed [98]. It has been shown [99] that in DNA translesion synthesis repair, DNA polymerases β or λ replace replicative DNA polymerase ε. It was demonstrated earlier [100,101,102,103] that DNA polymerase β interacts directly with PARP1 on the BER DNA substrates. PARP1 could be involved in switching BER synthesis from one polymerase to another.

**Figure 9:**
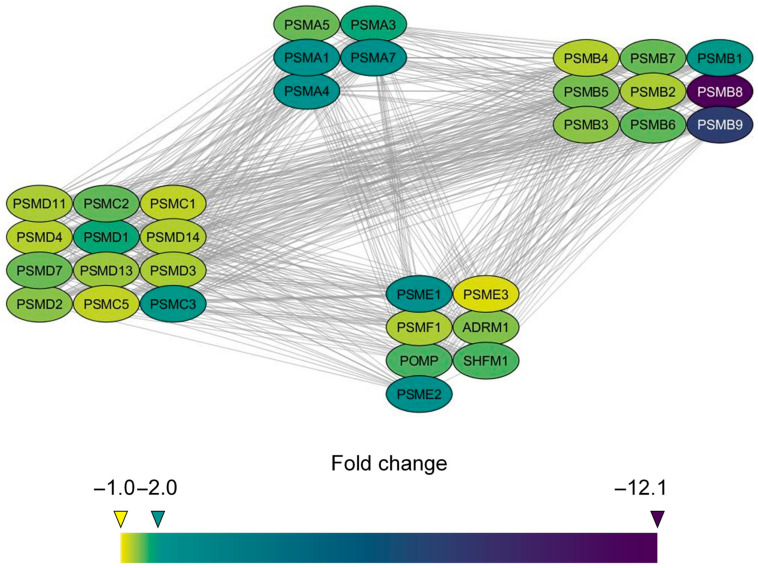
Network of protein-protein interactions of PARP1-dependent proteasome genes obtained using the STRING tool (minimum required interaction score 0.7). The color intensity of nodes depends on the fold change in the expression in PARP1 knockout cells compared to wild type cells. Rendered with Cytoscape v3.7.2. The group of genes at the top belongs to the α-ring, on the right, to the β-ring, on the left, to the 19S regulatory subunit, below—the rest of the genes.

A significant change in the level of expression of proteins responsible for the functioning of the proteasome is also of great interest (Table 2, Figure 9), and it was discovered for the first time for cells with the knockout of the *PARP1* gene. We note this fact because the elimination of TOP1cc by TDP1 requires the preliminary proteolytic degradation of the TOP1 protein globule, since TDP1 cannot hydrolyze the native TOP1cc conformation of covalent phosphotyrosyl bonds [25]. The proteolytic degradation of the TOP1 protein globule in TOP1cc can proceed through 26S proteasomal cleavage [59] or the elimination of TOP1cc with the SPRTN protease [63,64]. Both of these processes and TDP1 recruitment to the site of damage are under the control of PARylation of the TOP1cc and TDP1 [60,64,104]. Furthermore, it was shown in vivo that proteasomal degradation is significant to the processing of the PARP1 covalent DNA-protein crosslink (DPC) at the abasic site in double-stranded DNA in mouse fibroblasts [104]. Using a model DNA substrate mimicking the PARP1 DPC after proteasomal degradation, the authors found that repair was completed by the BER sub-pathway with TDP1 involvement. TDP1 binds to PARP1, and PARylation of TDP1 on its N-domain stabilizes TDP1 and promotes the recruitment of XRCC1 to TOP1cc damage sites and PNKP participation in DNA repair [18]. The authors of the work [59] note that TDP1 needs debulking of TOP1cc by the proteasome and SPRTN to gain access for TDP1 to the phosphotyrosyl bonds. We could see the most significant changes for only two subunits of the proteasome for PARP1-KO (Table 2), but we can also see that many genes have changed, although not significantly, and all these DEGs were down-regulated. We also observed DEGs in the signaling pathways associated with the proteasome in the experiments with all of the types of cell treatments that we had performed: Tpc, OL9-119, and a combination of Tpc with OL9-119 (Figure 5, Figure 6 and Figure 7, Tables S2–S4).

Trapped TOP1cc degraded by proteases to a short oligopeptide could be removed by TDP1. TDP1 is able to repair different 3′ end DNA adducts. Exposing cells to the action of TOP1 poison Tpc, we introduced damage to the DNA, and by adding the TDP1 inhibitor OL9-119, we did not allow TDP1 to restore the DNA. Next, after finding DEGs in PARP1-KO cells compared to wild type cells, we checked whether Tpc, OL9-119, and the combination of Tpc with OL9-119 influence the transcriptome of HEK293A wild type and PARP1-KO cells. We did not see any effects of these compounds on the transcriptome of PARP1-KO cells (Figure 5, Figure 6 and Figure 7). PARP1 is a key player in the repair of trapped TOP1cc by TDP1. PARP1-KO cells are more sensitive to Tpc than wild type cells. In our previous work [38], we checked this sensitivity after 72 h of treatment. In this work, we exposed cells to Tpc and other compounds for nearly 5 h to see the effect of the compound on the transcriptome in order to prevent the cells from dying. There was no effect in the first few hours, and then the cells probably died, for example, due to accumulated DNA damage and the complete dysregulation of the entire metabolism. Another assumption is that the knockout of the *PARP1* gene, under conditions of cells treatment with Tpc and OL9-119, does not have a cytotoxic, but, on the contrary, a cytoprotective function. PARP1 is involved in the development of oxidative stress, ER stress, autophagy, and cell death (parthanatos) [105,106]. In the absence of *PARP1* expression, these processes are impeded. Therefore, there is no detectable effect of the compounds on transcriptome changes in *PARP1*-knockout cells.

After treatment with the anticancer drug Tpc, we observed significant changes in WT cells, with the highest degree of DEG (Figure 5, Table S2) in the signaling pathways associated with processes including pathways in cancer, DNA replication, and repair, apoptosis, oxidative phosphorylation, and cell cycle. We found that DEGs in cancer-related signaling pathways, occurring in most pathways (Table S2), are responsible for the stress response (*GADD45*), regulate apoptosis (*BCL2*), NF-κB subunits and other genes associated with immune response, apoptosis, and the cell cycle. It should be noted that all cancer-associated signaling pathways in HEK293 cells are upregulated by Tpc treatment. The work [107] also shows that treatment of cells with Tpc causes an increase in *GADD45A* expression. It can be assumed that an increase in the expression of genes responsible for cell cycle arrest and apoptosis is another mechanism of the antiproliferative action of Tpc in addition to the suppression of TOP1 activity. In the work of [71], the authors also saw the changes in the transcriptome of MCF-7 tumor cells after Tpc treatment. They found that expression levels of the DNA repair genes, *MGMT* and *OGG*, were significantly decreased after Tpc treatment. In addition, the study [71] identified several genes, *FDXR*, *MSR*, *GSR*, and *GPx*, involved in the maintenance of cellular homeostasis due to increased ROS formation, were differentially expressed by Tpc, indicating a putative role of ROS and oxidative stress in Tpc cytotoxicity. This could be due to the parthanatos process, in which PAR accumulated in the nucleus goes to mitochondria, causing mitochondrial depolarization, which could lead to the release of the apoptosis-inducing factor AIF, which migrates into the nucleus and causes a caspase-dependent DNA fragmentation process [108]. Thus, it is likely that there is no effect of Tpc on PARP1-KO cells.

TDP1 appears as a target in cancer drug design owing to its ability to sensitize tumor cells to the action of TOP1 poisons and to break down various DNA adducts induced by chemotherapeutics. When we treated WT HEK293A cells with the effective TDP1 inhibitor OL9-119 compound, changes were found in the expression of proteins involved in different processes, the same as it was after Tpc treatment (Figure 5, Table S2), but DEGs associated with the spliceosome were found (Figure 6, Table S3). After treatment of these cells with a combination of Tpc and OL9-119, the highest degree of DEGs compared to only Tpc was observed (Table S4) in the signaling pathways associated with oxidative phosphorylation, spliceosome, proteasome, and ribosome. Oxidative phosphorylation is a cellular process that aims to form high-energy phosphate bonds in ATP, and it is the main and most efficient method of ATP synthesis under aerobic conditions. It is worthy of note that DEGs associated with this pathway, the same as proteasome and ribosome, were found after all the action types: PARP1-KO, and treatment with Tpc, OL9-119, or a combination of these compounds. The influence on the spliceosome was observed only under treatment of OL9-119 separately or with Tpc. There is no information about TDP1 in splicing, and the mechanism of the effect of this TDP1 inhibitor on the spliceosome requires further study.

## 4. Materials and Methods

### 4.1. CRISPR/Cas9-Directed Genome Editing

The knockout HEK293A cell line was generated as in [38] using the HEK293A WT (Thermo Fisher Scientific, Waltham, MA, USA) cell line. Briefly, cell clones with deletions in the region of constitutive proteins encoding exons (Supplementary Figure S1A) were obtained using the CRISPR/Cas9 method. The design of the sgRNAs for human *PARP1* gene knockout was performed using the Benchling CRISPR tool (https://www.benchling.com/, accessed on 9 November 2019) to delete the DNA sequence that includes 3–5 exons of the *PARP1* gene. The selected protospacers PARP1-gRNA1 (CTAGAACCTCCAATACCATG (TGG)) and PARP1-gRNA2 (GCAAGTGACCACAAAGGTGC (AGG) (PAM sequences in brackets)) were cloned in plasmid pSpCas9(BB)-2A-GFP (PX458) (Addgene #48138).

### 4.2. Knockout HEK293A Cells Generation

HEK293A cells were co-transfected with the generated plasmids pX458-PARP1-gRNA1 and pX458-PARP1-gRNA2 (0.25 µg of each) using a Lipofectamine 3000 Reagent (Thermo Fisher Scientific, Waltham, MA, USA). After transfection (48 h), the GFP-positive cells were sorted out and plated onto a 96-well plate, one cell per well. The growth medium contained DMEM/F12 (Gibco, Thermo Fisher Scientific, Waltham, MA, USA), 10% FBS (Biolot, Saint-Petersburg, Russia), 100 U/mL penicillin– streptomycin (Gibco, Thermo Fisher Scientific, Waltham, MA, USA), and 2 mM GlutaMAX (Gibco, Thermo Fisher Scientific, Waltham, MA, USA). Cell monoclones grew for two weeks in a 5% CO_2_ atmosphere at 37 °C. Clones were analyzed on deletions in the *PARP1* gene. Genome DNA was extracted from the cells, and DNA extract was used for PCR amplification of the target region with primers to detect the presence of deletions (PARP1-Del-F 5′-AGTGTGCCCTGCGTATTTGC-3′ and PARP1-Del-R 5′-CACAGGGATGAATCTTTCTGGTC-3′) and wild-type alleles (PARP1-In-F 5′-CGCTCCCTTGGTACCACATATG-3′ and PARP1-In-R 5′-GGCTTACTGACAGTCAGCGAAG-3′). Based on the analysis of PCR reaction products in 1% agarose gel stained with ethidium bromide, one cell clone (1A3) with homozygous deletions in the target gene was selected (Figure S1B).

For western blotting, the whole cell extracts (HEK293A WT and clone 1A3) were separated by Laemmli electrophoresis in 10% SDS-PAAG and were transferred on a nitrocellulose membrane (TransBlot Turbo, BIO-RAD, Hercules, CA, USA) using the semi-dry western blotting technique, and were probed with mouse antibody to PARP1 (Anti-Human PARP 556494, BD Biosciences, Bergen County, NJ, USA) or rabbit antibody to β-actin (Abcam 8226-100, Cambridge, UK). Blots were then probed with horseradish peroxidase-coupled goat anti-mouse antibody (1:15,000, made by L. Matveev, Biotechnological Laboratory, ICBFM SB RAS, Novosibirsk, Russia), and immunoreactivity was detected by chemiluminescence (Pierce ECL Western Blotting Substrate, Thermo Scientific, Waltham, MA, USA). The western blotting analysis showed the absence of PARP1 in the PARP1-knockout (PARP1-KO) cells (clone 1A3).

For immunofluorescence analysis, cells growing on 4-well plates were fixed in 4% formaldehyde for 10 min, permeabilized in a 0.5% Triton X-100 solution for 30 min, and incubated with a blocking buffer (10 mg/mL BSA in PBS). The above procedures were carried out at room temperature. Incubation with primary antibodies (Mouse Anti-Human PARP 556494, BD Biosciences, Bergen County, NJ, USA) was carried out overnight at +4 °C. The cells were incubated with secondary antibodies (Invitrogen Goat anti-Mouse IgG (H + L) Cross-Adsorbed Secondary Antibody, Alexa Fluor 488, Invitrogen, Waltham, MA, USA) in the dark at room temperature for 1–1.5 h. The cell nuclei were stained with DAPI. Preparations were analyzed on the Nikon Ti fluorescence microscope using NIS Elements software. An immunofluorescence analysis showed the absence of PARP1 in the PARP1-knockout (PARP1-KO) cells (clone 1A3).

### 4.3. Cell Culture Cytotoxicity Assay

HEK293A WT and PARP1-deficient (PARP1-KO) human cell lines were used to examine the cytotoxicity of the compounds by an xCELLigence DP Real Time Cell Analyzer (ACEA Biosciences, Santa Clara, CA, USA). A total of 2 × 10^4^ cells were plated onto E-Plate 16 PET (ACEA Biosciences, Santa Clara, CA, USA). Twenty-four h after cell passaging, the tested compounds were added to the medium, and the cell culture was monitored for 1–3 days. Control cells were grown in the complete growth medium containing 0.1% DMSO where indicated. Two parallel experiments were carried out for each compound concentration.

### 4.4. Total RNA Preparation

HEK293A WT and PARP1-KO cells were grown in a six-well plate for 20–21 h to double the number of cells in the well, the tested compounds were added to the medium, and the cell culture was grown for 5–5.5 h. For each sample there were four replicates. The RNA samples for the gene expression studies were prepared for the WT and PARP1-KO HEK293A cell lines with different treatments: (1) No treatment; (2) 0.1% DMSO; (3) 100 nM (for WT HEK293A) or 50 nM (for PARP1-KO HEK293A) Tpc + 0.1% DMSO; (4) 5 μM OL9-119 desolved in 0.1% DMSO; (5) 100 nM (for WT HEK293A) or 50 nM (for PARP1-KO HEK293A) Tpc + 5 μM OL9-119. Total RNA was isolated from cells lysed in Trizol with a PureLink RNA Mini Kit (Invitrogen, Waltham, MA, USA) and an On-column DNAse I Digestion Set (Sigma-Aldrich, St. Louis, MO, USA) according to the manufacturer’s instructions. The quantification of isolated RNA was performed on a Nanodrop 1000 spectrophotometer (Thermo Scientific, USA) and a Qubit 2.0 fluorometer with Qubit RNA and DNA High Sensitivity Assay Kits (Invitrogen, USA). The quality of RNA was estimated by means of measuring RNA Integrity Number (RIN) on an Agilent Bioanalyzer 2100 using an Agilent RNA 6000 Pico Kit (Agilent, Santa Clara, CA, USA).

### 4.5. Transcriptome Sequencing

To determine the effect of the chemical treatment of HEK293A WT and PARP1-KO cells on the level of expression of various genes, transcriptomic analysis of the samples in four technical replicates was performed. A NEBNext Poly(A) mRNA Magnetic Isolation Module (NEB, Ipswich, MA, USA) was used to isolate poly(A)+ RNA from total RNA. The quality of enriched poly(A)+ RNA was assessed on an Agilent Bioanalyzer 2100 using an Agilent RNA 6000 Pico Kit (Agilent, USA). Library preparation was performed with an MGIEasy RNA Directional Library Prep Set (MGI Tech Co., Ltd., Shenzhen, China) from poly(A)+ RNA. DNA libraries were sequenced with 100 bp paired-ends reagents on MGIseq 2000 (MGI Tech Co., Ltd., China), with a 30 million coverage per sample. All relevant procedures were performed in the SB RAS Genomics Core Facility (ICBFM SB RAS, Novosibirsk, Russia).

### 4.6. Differential Expression Analysis

The resulting reads were aligned to the human genome (hg38, ensembl v38.93) using STAR-2.7.8. Quality control of the reads was performed using FastQC, module infer_experiment.py of RSeQC, and module CollectRnaSeqMetrics from Picard. The results were compiled into a single report using the MultiQC package. The number of reads mapped to genes was also estimated using STAR, option-quantMode Gene-Counts.

The generated gene count table was analyzed using the DEseq2 package. Rows containing less than 10 counts were removed. A principal component analysis was performed after vst normalization. Differentially expressed genes were selected for further analysis at *p* < 0.01 after adjusting for multiple comparisons. A VolcanoPlot was generated using the EnhancedVolcano package. The fgsea package with the KEGG database was used to analyze signaling pathway enrichment.

## 5. Conclusions

In the present work, the effect of the chemical treatment of WT and PARP1-knockout (PARP1-KO) HEK293A cells on the level of expression of various genes was studied. The transcriptomic analysis of wild type and PARP1 knockout HEK293A cells treated with Tpc and TDP1 inhibitor OL9-119 alone and in combination showed that the largest number of differentially expressed genes were found in knockout cells. Topotecan and OL9-119 treatment gave rise to significantly less DEGs in WT cells, and negligible DEGs in PARP1-KO cells. A main part of the changes caused by PARP1-KO affected the synthesis and processing of proteins, cancer development, oxidative phosphorylation, and base excision repair. We discovered that the level of expression of genes of several key proteins participating in BER was reduced in the cells of the resulting cell line. Changes under the treatment with TOP1 and TDP1 inhibitors separately were found in the signaling pathways for the development of cancer, DNA repair, cell cycle control and apoptosis, and the proteasome. The drug combination resulted in DEG in the ribosome, proteasome, spliceosome, and oxidative phosphorylation.

## Figures and Tables

**Table 1:** DEGs involved in the process of DNA base excision repair in PARP1-KO cells vs. WT cells.

Name	Transcript.ID	log2FoldChange	*p*-Value	*p*-adj
*PARP1*	ENSG00000143799	−4.382	0.0	0.0
*NEIL1*	ENSG00000140398	−2.04	6.1 × 10^−17^	5.3 × 10^−15^
*POLE4*	ENSG00000115350	−1.559	2.8 × 10^−10^	7.4 × 10^−9^
*POLD2*	ENSG00000106628	−0.736	6.0 × 10^−9^	1.2 × 10^−7^
*SMUG1*	ENSG00000123415	−0.702	1.1 × 10^−5^	9.9 × 10^−5^
*FEN1*	ENSG00000168496	−0.433	2.0 × 10^−4^	0.001
*POLB*	ENSG00000070501	−0.566	4.0 × 10^−4^	0.002
*MPG*	ENSG00000103152	−0.896	5.9 × 10^−4^	0.003
*NEIL3*	ENSG00000109674	−0.535	0.004	0.017
*POLD4*	ENSG00000175482	−1.766	0.012	0.039

**Table 2:** DEGs involved in the process of proteasome in PARP1-KO cells vs. WT cells.

Name	Transcript.ID	log2FoldChange	*p*-Value	*p*-adj
*PSMA3*	ENSG00000100567	−0.83	7.4 × 10^−22^	6.7 × 10^−20^
*PSMD1*	ENSG00000173692	−0.834	3.5 × 10^−21^	3.0 × 10^−19^
*PSMC2*	ENSG00000161057	−0.647	3.4 × 10^−20^	2.7 × 10^−18^
*PSMC3*	ENSG00000165916	−0.967	3.1 × 10^−17^	1.8 × 10^−15^
*PSMB1*	ENSG00000008018	−0.968	2.8 × 10^−16^	1.4 × 10^−14^
*PSME1*	ENSG00000092010	−1.082	1.3 × 10^−15^	5.8 × 10^−14^
*PSMA4*	ENSG00000041357	−1.042	6.2 × 10^−15^	2.5 × 10^−13^
*PSMA7*	ENSG00000101182	−1.027	2.1 × 10^−12^	6.0 × 10^−11^
*PSME2*	ENSG00000100911	−1.129	5.5 × 10^−12^	1.5 × 10^−10^
*PSMA1*	ENSG00000129084	−1.057	1.0 × 10^−11^	2.6 × 10^−10^
*PSMD2*	ENSG00000175166	−0.533	1.7 × 10^−11^	4.1 × 10^−10^
*PSMA5*	ENSG00000143106	−0.624	2.0 × 10^−9^	3.3 × 10^−8^
*PSMF1*	ENSG00000125818	−0.395	3.3 × 10^−9^	5.3 × 10^−8^
*PSMB2*	ENSG00000126067	−0.409	9.0 × 10^−9^	1.3 × 10^−7^
*PSMD11*	ENSG00000108671	−0.41	1.7 × 10^−7^	1.9 × 10^−6^
*PSMB7*	ENSG00000136930	−0.629	5.3 × 10^−7^	5.4 × 10^−6^
*PSMB9*	ENSG00000240065	−2.945	6.5 × 10^−7^	6.5 × 10^−6^
*POMP*	ENSG00000132963	−0.709	1.2 × 10^−6^	1.2 × 10^−5^
*PSMD13*	ENSG00000185627	−0.464	1.7 × 10^−6^	1.6 × 10^−5^
*PSMB8*	ENSG00000204264	−3.595	2.9 × 10^−6^	2.5 × 10^−5^
*SEM1*	ENSG00000127922	−0.7	5.0 × 10^−6^	4.1 × 10^−5^
*PSMD7*	ENSG00000103035	−0.619	2.9 × 10^−5^	2.0 × 10^−4^
*PSMB3*	ENSG00000277791	−0.521	8.6 × 10^−5^	5.2 × 10^−4^
*PSMB6*	ENSG00000142507	−0.655	9.2 × 10^−5^	5.5 × 10^−4^
*PSMD3*	ENSG00000108344	−0.406	9.6 × 10^−5^	5.7 × 10^−4^
*PSMB5*	ENSG00000100804	−0.587	2.6 × 10^−4^	0.001
*PSMD4*	ENSG00000159352	−0.36	8.4 × 10^−4^	0.004
*PSMD14*	ENSG00000115233	−0.381	0.001	0.005
*PSMB4*	ENSG00000159377	−0.37	0.003	0.011
*PSMC1*	ENSG00000100764	−0.331	0.005	0.016
*ADRM1*	ENSG00000130706	−0.551	0.005	0.016
*PSME3*	ENSG00000131467	−0.196	0.006	0.02
*PSMC5*	ENSG00000087191	−0.314	0.011	0.031

## Data Availability

https://www.ncbi.nlm.nih.gov/geo/query/acc.cgi?acc=GSE218871 (accessed on 1 December 2022).

## Supplementary Materials

The following supporting information can be downloaded at: https://www.mdpi.com/article/10.3390/ijms24065148/s1.
