# Supplementary material for: Transcriptomic Analysis of CRISPR/Cas9-Mediated PARP1-Knockout Cells under the Influence of Topotecan and TDP1 Inhibitor
Source: Int J Mol Sci. 2023 Mar 7;24(6):5148. doi: 10.3390/ijms24065148 (PMC10049738; doi:10.3390/ijms24065148)
Supplement: Supplementary file 1 [file ijms-24-05148-s001.zip › ijms-2238446-supplementary.pdf]

# Transcriptomic Analysis of CRISPR/Cas9-Mediated PARP1-Knockout Cells under the Influence of Topotecan and TDP1 Inhibitor

**Nadezhda S. Dyrkheeva [1], Anastasia A. Malakhova [1,2], Aleksandra L. Zakharenko [1], Larisa S. Okorokova [3], Dmitriy N. Shtokalo [3,4], Sophia V. Pavlova [1,2], Sergey P. Medvedev [1,2], Suren M. Zakian [1,2], Anna A. Nushtaeva [1], Alexey E. Tupikin [1], Marsel R. Kabilov [1], Svetlana N. Khodyreva [1], Olga A. Luzina [5,*], Nariman F. Salakhutdinov [5] and Olga I. Lavrik [1,6]**

[1] Institute of Chemical Biology and Fundamental Medicine, Siberian Branch of the Russian Academy of Sciences, 8 Lavrentyeva Ave., 630090 Novosibirsk, Russia
[2] Federal Research Centre Institute of Cytology and Genetics, Siberian Branch of the Russian Academy of Sciences, 10 Lavrentyeva Ave., 630090 Novosibirsk, Russia
[3] AcademGene LLC, 6 Lavrentyeva Ave., 630090 Novosibirsk, Russia
[4] A.P. Ershov Institute of Informatics Systems SB RAS, 6 Lavrentyeva Ave., 630090 Novosibirsk, Russia
[5] N.N. Vorozhtsov Novosibirsk Institute of Organic Chemistry, Siberian Branch of the Russian Academy of Sciences, 9 Lavrentyeva Ave., 630090 Novosibirsk, Russia
[6] Department of Molecular Biology and Biotechnology, Novosibirsk State University, 630090 Novosibirsk, Russia
* Correspondence: luzina@nioch.nsc.ru

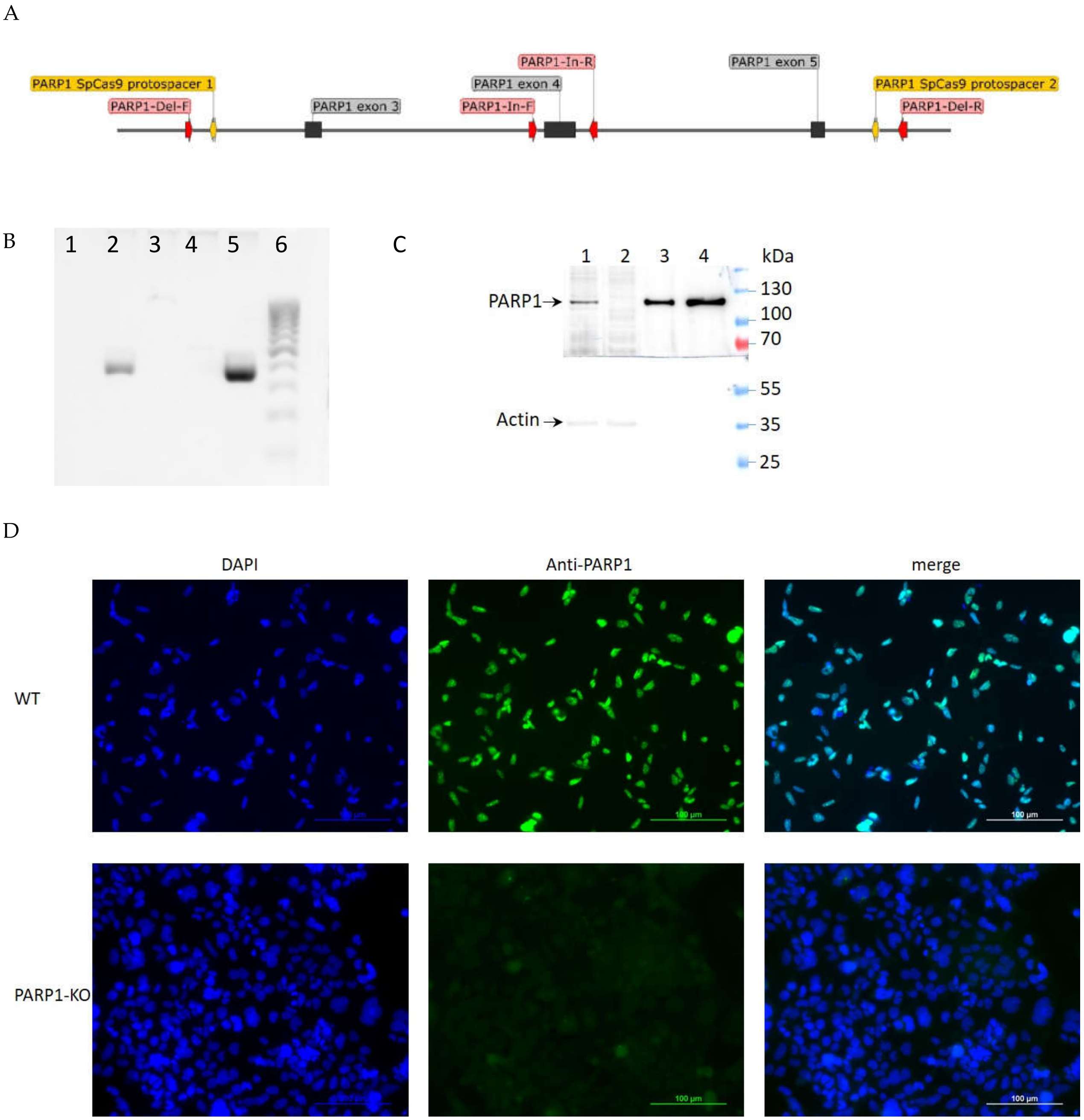


**Figure S1.** Scheme of deletion in *PARP1* gene (A). PCR analysis of CRISPR/Cas9-induced deletion (B): lanes: 1) $H_2O$; 2) PCR analysis of clone 1A3 genomic DNA with primers for detecting a deletion (PARP-Del-F and PARP-Del-R) in the PARP1 protein gene; 3) PCR analysis of clone 1A3 genomic DNA with primers for the detection of wild-type alleles (PARP-In-F and PARP-In-R); 4) PCR analysis of intact genomic DNA of HEK293A cells with deletion detection primers (PARP-Del-F and PARP-Del-R); 5) PCR analysis of intact genomic DNA of HEK293A cells with primers for the detection of wild-type alleles (PARP-In-F and PARP-In-R) 6) DNA ladder Step100 (Biolabmix, Novosibirsk, Russia). Western-blot analysis (C): whole cell extracts were separated by Laemmli electrophoresis in 10% SDS-PAAG and were transferred on nitrocellulose membrane using semi-dry Western Blotting technique, and were probed with mouse antibody to PARP1 (Anti-Human PARP 556494, BD Biosciences) or rabbit antibody to β-actin (Abcam8226-100). Lanes: 1) HEK293A WT cells; 2) HEK293A clone 1A3; 3) purified PARP1 (20 ng); 4) purified PARP1 (50 ng); protein ladder (Thermo Fisher Scientific). Immunofluorescence analysis (D): WT and clone 1A3 cells were fixed in 4% formaldehyde and stained with DAPI or incubated with primary anti-PARP1 antibodies. Preparations were analyzed on the Nikon Ti fluorescence microscope using NIS Elements software.

**Table S1.** PARP1 knockout effect on gene expression. Gene set enrichment analysis results: pathways significantly associated with differentially expressed genes.

| Pathway | *p*-Value | *p*-adj | NES | Number | Genes |
|---|---|---|---|---|---|
| Parkinson's disease | 5.2e-16 | 1.6e-13 | -2.579 | 109 | GNAI2, UCHL1, NDUFA4, SNCA, PARK7, NDUFV1, VDAC3, NDUFA8, NDUFC1, PRKACB, PINK1, UBB, COX8A, SDHD, UQCR10, NDUFS6, UBE2L3, SDHA, UBE2L6, NDUFS8, NDUFS5, COX6C, NDUFB4, UBA1, SLC25A4, COX7A2L, COX5B, NDUFA3, NDUFS3, COX7A2, COX5A, UQCRB, UQCR11, NDUFB2, UQCRQ, UQCRC1, NDUFA2, NDUFB8, NDUFA12, CASP3, VDAC2, NDUFA6, COX7B, NDUFA9, NDUFC2, COX4I1, NDUFV3, NDUFAB1, HTRA2, UBE2G2, PPIF, NDUFB10, COX6A1, PRKACA, NDUFA1, NDUFB1, SLC25A6, NDUFB3, COX6B1, NDUFB5, NDUFB6, NDUFS4 |
| Alzheimer's disease | 2.8e-14 | 4.3e-12 | -2.413 | 144 | BACE1, CAPN1, CDK5, PSEN2, PPP3CB, NDUFA4, RTN3, SNCA, PLCB3, CALM2, NDUFV1, CAPN2, APBB1, NDUFA8, NDUFC1, FADD, COX8A, SDHD, UQCR10, NDUFS6, TNFRSF1A, SDHA, BAD, NDUFS8, NDUFS5, COX6C, HSD17B10, NDUFB4, COX7A2L, COX5B, NDUFA3, NDUFS3, COX7A2, CALM3, FAS, COX5A, UQCRB, IDE, PPP3CA, UQCR11, NDUFB2, APH1A, UQCRQ, UQCRC1, NDUFA2, ATP2A3, NDUFB8, NDUFA12, CASP3, NDUFA6, COX7B, NDUFA9, NDUFC2, COX4I1, NDUFV3, NDUFAB1, NDUFB10, COX6A1, NDUFA1, NDUFB1, BID, NDUFB3, COX6B1, NDUFB5, CASP8, NDUFB6, NDUFS4, CACNA1F, MAPK3 |
| Oxidative phosphorylation | 5.6e-14 | 5.9e-12 | -2.552 | 98 | ATP6V0E2, PPA2, NDUFA4, TCIRG1, NDUFV1, COX15, NDUFA8, NDUFC1, PPA1, COX8A, SDHD, UQCR10, NDUFS6, SDHA, NDUFS8, NDUFS5, COX6C, ATP6AP1, NDUFB4, COX7A2L, COX5B, NDUFA3, NDUFS3, COX7A2, COX17, COX5A, UQCRB, UQCR11, NDUFB2, UQCRQ, UQCRC1, NDUFA2, NDUFB8, NDUFA12, NDUFA6, COX7B, NDUFA9, NDUFC2, COX4I1, ATP6V1H, NDUFV3, NDUFAB1, ATP6V0D1, ATP6V0E1, NDUFB10, COX6A1, NDUFA1, NDUFB1, NDUFB3, ATP6V0A4, COX6B1, NDUFB5, LHPP, NDUFB6, ATP6V1F, NDUFS4 |
| Lysosome | 1.5e-11 | 9.3e-10 | -2.318 | 121 | CTSD, SCARB2, ACP2, LAPTM4B, CTSH, PSAP, TCIRG1, CTSZ, ARSA, AGA, LIPA, TPP1, LAMP1, CTSV, SUMF1, LITAF, SORT1, CTSO, HEXA, SMPD1, LAPTM4A, HGSNAT, CTSB, CTSL, ATP6AP1, CTSA, LGMN, GM2A, FUCA1, PPT1, GNS, GLB1, NAGPA, CD63, AP1S1, AP1B1, NAGA, PPT2, MANBA, ATP6V1H, ATP6V0D1, ARSB, CTNS, AP1G2, NEU1, GNPTG, AP3M1, ATP6V0A4, PLA2G15, NAGLU, DNASE2, ABCA2, AP3S2, IDUA |
| Proteasome | 1.2e-11 | 9.3e-10 | -2.496 | 43 | PSMA3, PSMD2, PSMD1, PSMA4, PSMC3, PSMA7, PSMC2, PSMB1, PSME1, PSME2, PSMA1, PSMF1, PSMB2, PSMD3, PSMA5, PSMB6, PSMD11, PSMD13, PSMB9, PSMB7, POMP, SEM1, PSMB8, PSMB5, PSMD7, ADRM1, PSMB3, PSMB4, PSMD4, PSMD14, PSMC5, PSME3, PSMC1 |
| Ribosome | 8.3e-07 | 4.3e-05 | -1.98 | 129 | MRPL23, MRPL11, MRPS16, RPS25, RPL7A, RPL34, MRPS9, MRPL16, MRPL15, MRPL1, RPS3A, MRPL28, MRPL35, MRPS18C, MRPL30, MRPL27, RPS24, MRPS11, MRPL33, RPS27A, MRPL20, RPL27A, RPS3, MRPL21, RPL26L1, MRPL19, RPSA, RPS20, MRPS7, FAU, MRPL12, MRPL14, RPS13, RPLP1, RPS7, MRPS2, MRPL3, RPL7, RPL23A, RPL9, RPL36AL, MRPS6, MRPS14, RPL31, MRPS18A, RPL35A, MRPS15, RPL29, MRPS5, RPS26, MRPL36, RPL19, RPS9, RPL3, RPL6 |

| | | | | | |
|---|---|---|---|---|---|
| Oocyte meiosis | 2.0e-06 | 7.7e-05 | -1.979 | 109 | CCNB1, PPP3CB, PPP1CA, CALM2, YWHAZ, YWHAQ, CAMK2G, BUB1, CDC20, CUL1, PRKACB, MAD2L1, CCNB2, YWHAB, AURKA, PPP2R1A, PPP2R5D, ADCY6, RPS6KA2, PPP2R1B, PTTG1, CALM3, CDC16, PPP3CA, ANAPC2, PLK1, CDC25C, PPP2R5B, MAD2L2, RBX1, YWHAE, PPP2CB, SKP1, AR, MAPK12, RPS6KA1, BTRC, PRKACA, ADCY3, CDK1, PPP1CB, ANAPC10, CDC26, CAMK2D |
| Protein processing in endoplasmic reticulum | 1.8e-06 | 7.7e-05 | -1.866 | 157 | VCP, GANAB, PDIA4, CKAP4, CAPN1, PDIA6, MBTPS2, STT3A, HSPA8, HYOU1, RPN2, CALR, BCAP31, UBE2D4, UBE2D3, CAPN2, DNAJB12, NSFL1C, CUL1, SYVN1, PDIA3, MAN1B1, FBXO6, OS9, PREB, HSPA1B, RRBP1, ATF6B, SEC61A1, SEC61G, P4HB, LMAN2, TRAM1, HSP90B1, STUB1, SEC61B, SSR4, FBXO2, ERLEC1, UBE4B, SAR1A, SSR2, MAPK8, SEC24C, MOGS, SEC31A, RBX1, WFS1, TRAF2, DAD1, DDOST, SKP1, RNF5, SEC24D, UBE2G2, RPN1, SSR3, ERO1B, MBTPS1, UGGT1, DERL2, DNAJB11, EIF2AK1, SELENOS, DNAJC5, BAX |
| Huntington's disease | 4.7e-06 | 1.6e-04 | -1.773 | 169 | NDUFA4, POLR2L, PLCB3, POLR2G, NDUFV1, DCTN1, VDAC3, AP2M1, NDUFA8, NDUFC1, COX8A, SDHD, UQCR10, NDUFS6, SDHA, NDUFS8, NDUFS5, COX6C, POLR2J, NDUFB4, SLC25A4, COX7A2L, SOD1, COX5B, NDUFA3, NDUFS3, COX7A2, COX5A, UQCRB, UQCR11, NDUFB2, POLR2E, UQCRQ, UQCRC1, NDUFA2, CREB3, NDUFB8, NDUFA12, CASP3, VDAC2, NDUFA6, COX7B, NDUFA9, NDUFC2, COX4I1, DNAH5, NDUFV3, NDUFAB1, PPIF, DCTN2, NDUFB10, COX6A1, AP2B1, NDUFA1, AP2A2, NDUFB1, SLC25A6, GPX1, TAF4, BAX, NDUFB3, COX6B1, NDUFB5, CASP8, NDUFB6, IFT57, NDUFS4, AP2A1, POLR2K |
| Vibrio cholerae infection | 5.2e-06 | 1.6e-04 | -2.073 | 48 | PDIA4, ATP6V0E2, TCIRG1, KCNQ1, PRKACB, ARF1, KDELR1, GNAS, SEC61A1, ATP6AP1, SEC61G, SEC61B, ACTB, ACTG1, ATP6V1H, ATP6V0D1, ATP6V0E1, PRKACA, ADCY3, KDELR2, ATP6V0A4, ATP6V1F |
| Fatty acid degradation | 1.2e-05 | 2.4e-04 | -2.115 | 37 | ADH5, HADH, ECHS1, HADHA, ACAT1, ALDH2, CPT2, ACSL1, ACADVL, ECI2, ALDH3A2, ACAA1, ACOX3, HADHB, CPT1A, ACAA2, ECI1 |
| N-Glycan biosynthesis | 9.1e-06 | 2.4e-04 | -2.04 | 47 | GANAB, STT3A, RPN2, DPAGT1, DOLK, ALG8, MAN1B1, MGAT3, B4GALT2, MOGS, ALG1, DAD1, DDOST, RPN1, MGAT4B, DPM2, MGAT1, FUT8, ALG6, ALG9, ST6GAL1 |
| Phagosome | 1.1e-05 | 2.4e-04 | -1.85 | 135 | HLA-B, ATP6V0E2, HLA-C, TUBB4B, C1R, TUBB, TCIRG1, HLA-A, CALR, TAP1, TUBA1C, HLA-DPA1, RAB5B, TUBA1B, ITGB5, LAMP1, RAC1, HLA-DQB1, SEC61A1, MRC2, CTSL, ATP6AP1, SEC61G, SEC61B, ACTB, VAMP3, PLA2R1, COLEC12, HLA-DPB1, ACTG1, RAB5C, ATP6V1H, ATP6V0D1, ATP6V0E1, DYNC1I2, RAB7B, HLA-DRB1, TUBB2A, ATP6V0A4, ATP6V1F, ITGA5, ITGB3 |
| Retrograde endocannabinoid signaling | 9.3e-06 | 2.4e-04 | -1.885 | 129 | GNAI2, NDUFA4, PLCB3, NDUFV1, NDUFA8, NDUFC1, PRKACB, NDUFS6, NDUFS8, NDUFS5, ADCY6, NDUFB4, MAPK11, NDUFA3, NDUFS3, NDUFB2, GNB2, MAPK8, DAGLA, NDUFA2, GABRA3, NDUFB8, NDUFA12, NDUFA6, NDUFA9, NDUFC2, FAAH, NDUFV3, NDUFAB1, GNG11, MAPK12, GABRB3, NDUFB10, PRKACA, NDUFA1, ADCY3, NDUFB1, NDUFB3, GNGT1, NDUFB5, NDUFB6, GNG5, NDUFS4, CACNA1F, MAPK3 |
| Ribosome biogenesis in eukaryotes | 9.8e-06 | 2.4e-04 | 1.919 | 78 | GNL3, GTPBP4, WDR43, RIOK1, WDR75, WDR3, LSG1, REXO1, UTP4, NVL, WDR36, NOP58, DKC1, XRN1, TCOF1, UTP14A, NXF1, NMD3, GNL2, RIOK2, CSNK2A1, UTP18, NXF3, RCL1, GNL3L, NOP56, UTP14C, MDN1, EFL1, FCF1, RPP38, NXT1, CSNK2A2, UTP15 |

| | | | | | |
|---|---|---|---|---|---|
| MicroRNAs in cancer | 4.8e-05 | 8.9e-04 | 1.688 | 165 | VEGFA, DNMT1, DDIT4, PIM1, CDC25A, HMOX1, IRS2, SLC7A1, ZEB1, CCND2, MDM4, E2F2, MCL1, CRKL, VIM, BRCA1, TNXB, MMP16, PTGS2, SPRY2, IGF2BP1, CDKN1A, KRAS, EP300, MYC, GLS, RASSF1, NOTCH3, ROCK1, MDM2, APC, ABCB1, BCL2, RPS6KA5, E2F3, IRS1, MAP2K1, TNC, RAF1, SOX4, APC2, PIK3CA, ZEB2, CRK, TIMP3, DICER1, MIRLET7D |
| Non-alcoholic fatty liver disease (NAFLD) | 4.3e-05 | 8.5e-04 | -1.786 | 135 | NDUFA4, RXRA, NDUFV1, NDUFA8, NDUFC1, RAC1, COX8A, SDHD, UQCR10, NDUFS6, AKT1, MAP3K11, TNFRSF1A, SDHA, NDUFS8, NDUFS5, COX6C, NDUFB4, COX7A2L, COX5B, NDUFA3, NDUFS3, COX7A2, FAS, COX5A, UQCRB, NFKB1, UQCR11, NDUFB2, TGFB1, AKT3, MAPK8, UQCRQ, CDC42, UQCRC1, NDUFA2, NDUFB8, NDUFA12, CASP3, TRAF2, NDUFA6, COX7B, NDUFA9, NDUFC2, COX4I1, NDUFV3, NDUFAB1, IKBKB, NDUFB10, |
| Glutathione metabolism | 6.8e-05 | 0.001 | -1.98 | 44 | GSTP1, GPX3, MGST2, SMS, G6PD, IDH1, GSTO1, TXNDC12, IDH2, LAP3, GSTK1, GGT1, GSTO2, GSS, GPX1, GSR, PGD, GGT7, GSTM4, GSTA4, RRM1, GSTM3, MGST1, GPX4, GGT6, GGCT |
| Antigen processing and presentation | 1.0e-04 | 0.002 | -1.903 | 60 | HLA-B, HLA-C, HSPA8, TAPBP, HLA-A, CALR, TAP1, HLA-DPA1, PSME1, PSME2, PDIA3, HSPA1B, CTSB, HLA-DQB1, CTSL, LGMN, B2M, RFXANK |
| Axon guidance | 1.2e-04 | 0.002 | -1.677 | 170 | CFL1, GNAI2, CDK5, PPP3CB, SEMA4F, RAC2, SSH3, SMO, RHOD, CAMK2G, UNC5B, NCK2, NRP1, RAC1, GDF7, ILK, HRAS, EFNA3, EFNB3, WNT5B, NTN1, PAK1, PPP3CA, BMPR1B, FZD3, ABLIM2, UNC5C, CDC42, EPHB2, DPYSL5, DPYSL2, RHOA, SEMA4B, SEMA4G, EPHA1, LIMK1, RAC3, BOC, NTN4, PLXNB1, PRKCZ, CAMK2D, LIMK2, RGS3, PLXNA3, EFNA4, BMPR2, SEMA3B, EPHB3, TRPC3, ROBO3, EPHA6, MAPK3, NFATC4, SLIT2, WNT5A, NTN3, PARD6A, PLXNA1, SRC, ROBO1, PIK3CD |
| Transcriptional misregulation in cancer | 1.6e-04 | 0.002 | 1.634 | 159 | GADD45B, GADD45A, ETV1, DDIT3, PER2, NUPR1, CEBPB, ZEB1, CCND2, CXCL8, CCNT1, DDX5, CCNT2, DUSP6, BCL6, ETV5, RARA, KDM6A, PRCC, SP1, ELK4, MEF2C, CD14, SIX1, CDKN1A, NR4A3, ETV4, NFKBIZ, MYC, TCF3, PAX3, DOT1L, POLK, SIX4, JMJD1C, MDM2, GADD45G, CD86, ATF1, PTK2, MITF |
| Endocytosis | 2.1e-04 | 0.003 | -1.524 | 241 | HLA-B, HLA-C, ARFGAP2, HSPA8, CHMP3, ARF3, GRK2, HLA-A, RAB5B, AP2M1, ARAP1, CHMP6, VPS26B, UBB, ARF1, RAB4A, SNF8, ARF5, ARRB1, FGFR3, HRAS, LDLRAP1, HSPA1B, CAPZB, ASAP3, VPS26A, PSD4, FOLR1, AGAP3, ZFYVE27, TSG101, FGFR2, CAV2, RAB31, CAV1, CDC42, ASAP2, RAB11A, RHOA, RAB11FIP3, SNX1, VPS37C, ARPC2, CHMP2A, RAB5C, CAPZA2, MVB12A, VPS25, VPS36, VPS4A, SNX12, VPS29, AP2B1, GRK4, AP2A2, EHD3, CHMP4C, EHD1, WASHC2C, CYTH2, ARPC4, PRKCZ, RAB11FIP1, PLD2, RAB22A, ARPC3, BIN1, CHMP4B, WASHC2A, PIP5K1C, ARPC1A, RAB8A, CHMP7, SMAD2, AP2A1, WIPF3, CYTH3, MVB12B, CHMP5, RAB11B, LDLR, PARD6A, HLA-E, CHMP1A, SRC, GRK5 |
| Gastric acid secretion | 2.7e-04 | 0.004 | -1.787 | 68 | GNAI2, KCNQ1, PLCB3, CALM2, SLC4A2, CAMK2G, CCKBR, PRKACB, GNAS, ADCY6, CALM3, ACTB, SSTR2, ATP1B2, PRKACA, ADCY3, CA2, CAMK2D |
| Peroxisome | 2.7e-04 | 0.004 | -1.834 | 83 | CAT, CRAT, PEX2, ACSL1, PRDX1, PEX19, GNPAT, ECI2, PEX10, SOD1, IDH1, PMVK, ABCD1, ACAA1, IDH2, ACOX3, GSTK1, PRDX5, MVK, PEX11B, SCP2, PEX7, PECR, CROT, ABCD4, SLC25A17, PEX26, PEX16 |
| Sphingolipid signaling pathway | 4.1e-04 | 0.005 | -1.681 | 116 | CTSD, GNAI2, RAC2, PPP2R2C, PLCB3, SGPL1, RAC1, HRAS, AKT1, SMPD1, TNFRSF1A, PPP2R1A, PPP2R5D, PPP2R1B, PTEN, MAPK11, NFKB1, GNA12, AKT3, MAPK8, DEGS1, PPP2R5B, CERS5, RHOA, TRAF2, |

| | | | | | |
|---|---|---|---|---|---|
| | | | | | PPP2R3B, PPP2CB, CERS2, SGMS1, MAPK12, CERS1, SPTLC3, RAC3, BID, PRKCZ, ADORA1, NOS3, BAX, PLD2 |
| Citrate cycle (TCA cycle) | 5.8e-04 | 0.006 | -1.851 | 29 | MDH1, PDHB, SDHD, SDHA, IDH1, OGDH, SUCLG1, IDH2, DLAT, MDH2, FH, IDH3G, CS, ACO2, PDHA1, IDH3A |
| Focal adhesion | 4.9e-04 | 0.006 | -1.531 | 197 | LAMB3, COL6A1, VEGFB, PARVA, COL2A1, RAC2, PPP1CA, LAMB2, CAPN2, COL6A2, COL4A2, ITGA3, ITGB5, RAC1, ILK, HRAS, PARVB, AKT1, BAD, LAMA5, COL1A1, PTEN, PDGFA, PDGFC, ITGA7, CAV2, PAK1, ACTB, CAV1, COL4A1, AKT3, MAPK8, CDC42, CCND1, ERBB2, COL9A2, RHOA, COL4A5, ACTG1, VAV2, ITGB4, SHC1, EGF, MYL9, MYL12B, PXN, LAMB1, RAC3, FLNA, PPP1CB, COL4A4, VAV1, ITGB8, PIP5K1C, FLT1, ITGA5, ITGB3, MAPK3, COL9A3, FN1, PDGFD, ITGA11, TLN1, LAMC3, BIRC2, VCL, SRC |
| Glycosaminoglycan biosynthesis - chondroitin sulfate / dermatan sulfate | 6.2e-04 | 0.006 | -1.933 | 20 | B3GALT6, CHPF2, B3GAT3, CHST14, CHST15, CHPF, B4GALT7, CHST11, CSGALNACT1, CHST12, CHST3, UST, XYLT2, CHSY1, CHST7 |
| Protein export | 4.9e-04 | 0.006 | -1.901 | 23 | SPCS2, SRP9, SEC61A1, SEC61G, SEC61B, SPCS3, SEC11C, SEC11A, SRP72, SPCS1, SRP14, IMMP2L, OXA1L |
| Tryptophan metabolism | 6.4e-04 | 0.006 | -1.85 | 37 | HADH, ECHS1, CAT, HADHA, ACAT1, ALDH2, MAOB, OGDH, CYP1B1, ALDH3A2 |
| Fatty acid elongation | 7.0e-04 | 0.007 | -1.866 | 25 | HADH, ECHS1, HADHA, HSD17B12, PPT1, HACD4, HADHB, ELOVL6, PPT2, HACD3, ELOVL1, ACAA2, HACD1 |
| Progesterone-mediated oocyte maturation | 8.5e-04 | 0.008 | -1.701 | 88 | CCNB1, GNAI2, CCNA2, BUB1, PRKACB, MAD2L1, CCNB2, AKT1, CDC25B, AURKA, ADCY6, RPS6KA2, MAPK11, CDC16, AKT3, MAPK8, ANAPC2, PLK1, CDC25C, CCNA1, MAD2L2, PDE3B, MAPK12, RPS6KA1, PRKACA, ADCY3, CDK1, ANAPC10, CDC26, ARAF |
| RNA transport | 8.0e-04 | 0.008 | 1.548 | 162 | EIF4A2, RNPS1, EIF3H, DDX39B, XPOT, EIF3D, EIF2S1, EIF5, EIF3A, EIF4B, NUP153, NUP58, TPR, PNN, DDX20, RAE1, CASC3, PABPC4, SEH1L, NUP155, NXF1, NMD3, EIF2B2, NUP107, NUP50, NUP214, NXF3, NUP62, THOC1, UPF2, PRMT5, THOC2, EIF4G1, NUP205, SRRM1, CYFIP1, EIF1B, NUP98, KPNB1, NUP43, EIF4A1, EEF1A2, MAGOH, EIF1, RPP38, RANBP2, NUP210L, CYFIP2, EIF3C, NUP35, SMN2, SMN1, NXT1, GEMIN5, NUP85, PHAX, UPF1, NCBP1, FXR1, SENP2, TGS1 |
| Base excision repair | 0.001 | 0.01 | -1.799 | 33 | PARP1, NEIL1, POLE4, POLD2, SMUG1, FEN1, POLB, MPG, NEIL3, POLD4, PARP3, NTHL1, LIG3, HMGB1 |
| Metabolism of xenobiotics by cytochrome P450 | 0.001 | 0.01 | -1.786 | 40 | ADH5, GSTP1, MGST2, EPHX1, CYP1B1, GSTO1, GSTK1, CBR1, GSTO2, CBR3, GSTM4, GSTA4, ALDH3B1, GSTM3, MGST1, ALDH1A3 |

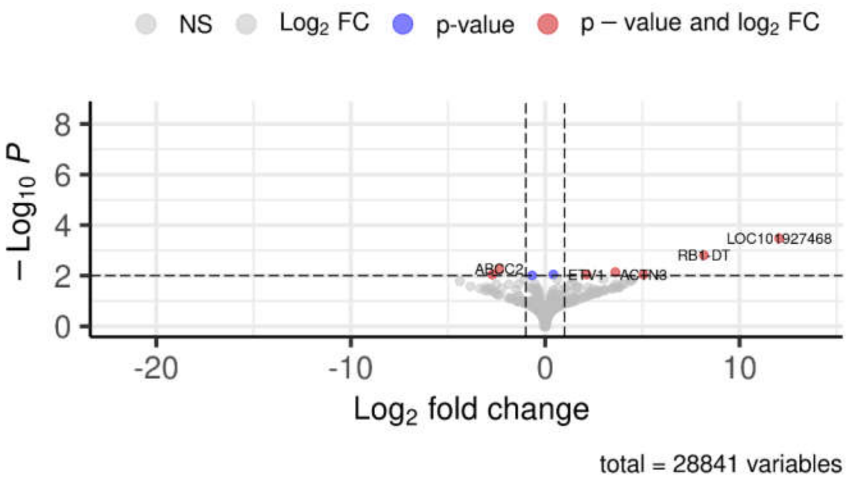


**A**

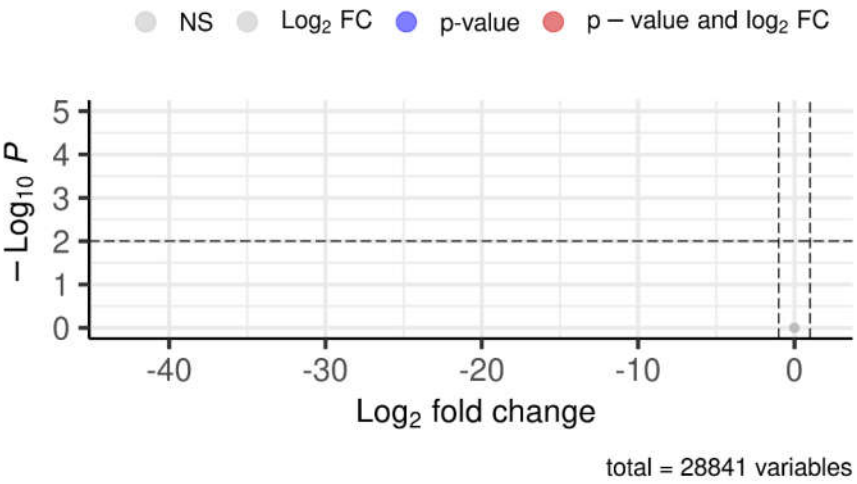


**B**

**Figure S2.** DMSO effect on WT (A) and PARP1-KO (B) cells. Volcano Plot for differential gene expression in cells treated with 0.1% DMSO vs untreated cells.

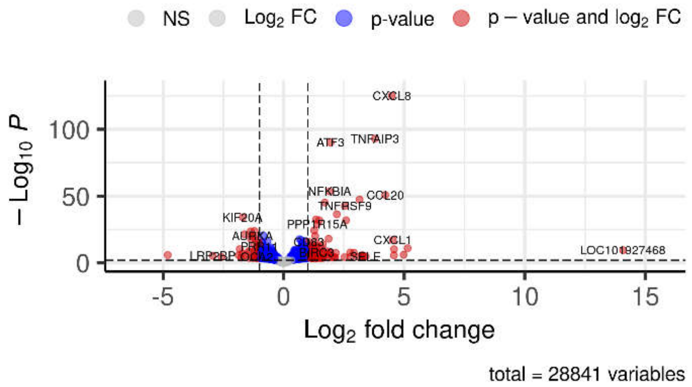


**A**

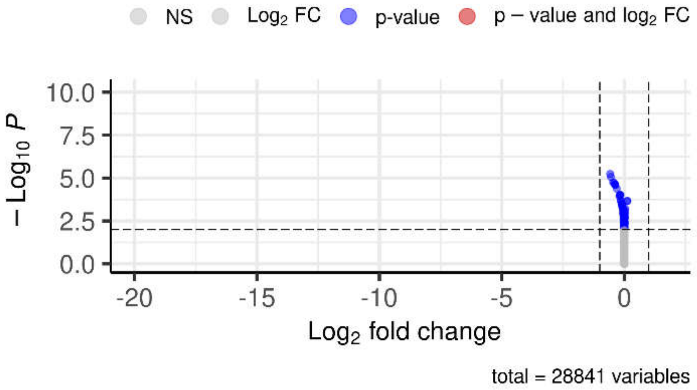


**B**

**Figure S3.** Topotecan effect on A) WT HEK293A cells treated with 100 nM Tpc vs untreated cells; B) PARP1-KO HEK293A cells treated with 50 nM Tpc vs untreated cells. Volcano Plots for differential gene expression under the influence of topotecan.

**Table S2.** Gene set enrichment analysis results of topotecan effect on gene expression: pathways significantly associated with differentially expressed genes upon treatment with topotecan plus DMSO or only DMSO (WT HEK293A cells treated with 100 nM Tpc + 0.1% DMSO vs cells treated with 0.1% DMSO).

| Pathway | *p*-Value | *p*-adj | NES | Number | Genes |
|---|---|---|---|---|---|
| Ribosome | 7.3e-24 | 2.3e-21 | -2.865 | 129 | MRPS9, RPL35, RPS27L, RPL7A, RPL19, RPL29, MRPL33, RPL38, MRPL23, UBA52, RPL24, RPLP0, FAU, RPS12, RPL27A, RPS3A, RPL35A, RPL36AL, RPS4X, RPL5, RPS19, RPL22, MRPL11, MRPL14, RPS25, RPS8, RPS6, RPL21, RPL37, RPS14, RPL3, RPS24, RPS7, RPL11, MRPL9, RPL23A, RPL31, RPL23, RPL13, RPS5, RPL34, RPL41, RPL6, RPL12, RPS11, RPL14, RPS2, RPS27A, MRPL27, RPL27, RPSA, RPL36, RPS18, RPLP1, RPS26, RPL13A, RPL37A, RPS27, RPL15, MRPL1, MRPL12, RPL10, RPL4, MRPL13, MRPL4, RPLP2, RPS23, RPS13, RPL8, RPL18, RPS20, RPL9, RPL10A, RPL32, MRPL28, RPL7, RPS16, MRPS14, RPS15A, MRPL35, MRPL21, RPL39, MRPS18A, MRPS5, MRPL24, RPS3, MRPL30 |
| Oocyte meiosis | 1.3e-14 | 2.0e-12 | -2.513 | 109 | CCNB1, CDC20, PLK1, AURKA, BUB1, CCNB2, CALM2, CDK1, PTTG1, PPP2R5C, YWHAE, PPP3CB, PPP2R5E, CDC25C, YWHAB, YWHAZ, PPP3CA, CUL1, PPP2R5D, SPDYA, CAMK2D, ANAPC5, CALM3, ANAPC11, ANAPC10, IGF1R, PPP2R1A, PPP1CB, REC8, ADCY6, SMC1B, PRKACB, RBX1, MAD2L2, PPP1CA, YWHAQ, MAD2L1, SKP1, PPP2CA, CDC26, ESPL1, BTRC, CDC16 |
| TNF signaling pathway | 8.5e-14 | 8.9e-12 | 2.517 | 106 | TNFAIP3, NFKBIA, CCL20, JUN, CXCL1, CREB5, JUNB, CXCL10, CXCL2, NFKB1, BIRC3, CXCL3, ICAM1, RELA, SELE, BIRC2, MAP3K14, CEBPB, LIF, TNF, CASP3, MAP3K8, TRAF1, FOS, CSF1, CHUK, CCL2, BCL3, CREB1, FAS, CASP10, JAG1, PIK3R1, VCAM1, TRAF3 |
| IL-17 signaling pathway | 5.9e-13 | 3.7e-11 | 2.527 | 85 | CXCL8, TNFAIP3, NFKBIA, CCL20, JUN, CXCL1, CXCL10, CXCL2, NFKB1, CXCL3, FOSL1, RELA, CEBPB, TNF, CASP3, JUND, TRAF4, FOS, TRAF3IP2, CHUK, CCL2, FOSB |
| Oxidative phosphorylation | 4.7e-13 | 3.7e-11 | 2.479 | 98 | NDUFB4, ATP6V0E2, NDUFAB1, COX7A2, ATP6V1H, NDUFA9, UQCR11, ATP6V1E1, NDUFS5, NDUFB10, ATP6V0E1, COX7B, UQCRQ, NDUFB3, NDUFA12, NDUFA3, COX5A, COX6B1, COX6C, NDUFA4, PPA2, NDUFB5, COX4I1, NDUFB9, NDUFS4, UQCR10, NDUFS6, NDUFB2, TCIRG1, NDUFA2, COX7C, COX5B, SDHC, UQCRH, UQCRC2, NDUFA8, COX17, NDUFB6, NDUFC1, SDHB, NDUFB11, NDUFV1, ATP6V0A4, NDUFB1, COX6A1, NDUFS8, ATP6V0D1, NDUFA1, UQCRC1, NDUFA5, COX8A, ATP6V1G1, NDUFA6, NDUFA10 |
| NF-kappa B signaling pathway | 1.8e-12 | 8.9e-11 | 2.479 | 86 | CXCL8, TNFAIP3, NFKBIA, RELB, PLAU, GADD45B, NFKB2, CXCL2, NFKB1, BIRC3, ICAM1, RELA, TICAM1, CCL4, BIRC2, MAP3K14, TNF, BCL2, TRAF1, CHUK, PIAS4, BTK, TIRAP, ZAP70, TRAF6, VCAM1, TRAF3, MALT1, BCL10, LCK |
| Parkinson's disease | 2.0e-12 | 8.9e-11 | -2.396 | 109 | NDUFB4, UBE2L3, NDUFAB1, COX7A2, NDUFA9, UQCR11, NDUFS5, NDUFB10, COX7B, GNAI2, SNCA, UQCRQ, NDUFB3, UBE2L6, NDUFA12, NDUFA3, COX5A, PARK7, COX6B1, COX6C, UBB, NDUFA4, NDUFB5, GNAI3, COX4I1, NDUFB9, NDUFS4, UQCR10, NDUFS6, UCHL1, NDUFB2, VDAC1, NDUFA2, PRKACB, COX7C, COX5B, SDHC, UBE2G1, UQCRH, PRKN, UQCRC2, NDUFA8, NDUFB6, NDUFC1, SDHB, VDAC3, NDUFB11, NDUFV1, NDUFB1, COX6A1, NDUFS8, UBE2G2, NDUFA1, UQCRC1, NDUFA5, COX8A, NDUFA6, GNAL, NDUFA10, VDAC2, SLC25A5, COX7A2L, GNAI1 |
| Alzheimer's disease | 3.9e-11 | 1.5e-09 | -2.261 | 144 | CALM2, NDUFB4, PPP3CB, ATF6, NDUFAB1, GSK3B, COX7A2, NDUFA9, PPP3CA, CACNA1F, UQCR11, NDUFS5, NDUFB10, COX7B, SNCA, UQCRQ, NDUFB3, RTN3, PLCB1, NDUFA12, GNAQ, HSD17B10, NDUFA3, |

| | | | | | |
|---|---|---|---|---|---|
| | | | | | COX5A, COX6B1, COX6C, ATP2A1, NDUFA4, GAPDH, CALM3, NDUFB5, CDK5, COX4I1, NDUFB9, NDUFS4, UQCR10, NDUFS6, CAPN2, APBB1, MME, NDUFB2, NDUFA2, CAPN1, COX7C, COX5B, SDHC, UQCRH, UQCRC2, NDUFA8, NDUFB6, NDUFC1, SDHB, BACE1, NDUFB11, NDUFV1, PSENEN, NDUFB1, COX6A1, NDUFS8, NDUFA1, UQCRC1, NDUFA5, COX8A, CACNA1S |
| NOD-like receptor signaling pathway | 5.0e-10 | 1.7e-08 | 2.19 | 144 | CXCL8, TNFAIP3, NFKBIA, JUN, CXCL1, GABARAPL1, CXCL2, NFKB1, BIRC3, CXCL3, RIPK2, RELA, TICAM1, DHX33, BIRC2, TNF, IRF7, MFN2, BCL2, OAS3, ANTXR2, CHUK, CCL2, TP53BP1, GBP1, PANX1, NLRX1, ATG12, TRAF6, OAS2, STAT1, NAMPT, TRIP6, TRAF3 |
| Progesterone-mediated oocyte maturation | 9.2e-09 | 2.9e-07 | -2.261 | 88 | CCNB1, PLK1, AURKA, BUB1, CCNA2, CCNB2, CDK1, CDC25C, CDC25B, AKT3, HSP90AA1, GNAI2, SPDYA, PIK3CB, ANAPC5, CCNA1, HSP90AB1, ANAPC11, ANAPC10, KIF22, GNAI3, IGF1R, ADCY6, MAPK11, PRKACB, MAD2L2, MAD2L1, PDE3B, CDC26, CDC16, CDC27, ARAF, RPS6KA1, MAD1L1, SPDYC, ADCY3 |
| Protein processing in endoplasmic reticulum | 1.0e-08 | 2.9e-07 | -2.046 | 157 | DNAJB1, HSPA1B, HSPA8, HSPA5, ATF6, VCP, BCAP31, UBE2D2, DNAJC1, RAD23B, SEC61G, CUL1, RPN1, PDIA3, HSP90AA1, HSP90B1, ERLEC1, RPN2, ERO1A, CALR, SEC63, STT3B, SSR2, NSFL1C, HSP90AB1, DAD1, RNF5, SSR3, PDIA6, STUB1, DNAJC3, SSR4, SIL1, CAPN2, BAX, SEC61B, OS9, SEC23B, RBX1, CAPN1, TUSC3, FBXO2, LMAN2, UBE2G1, TRAM1, SKP1, PRKCSH, PRKN, UBE2D4, SEC13, HSPBP1, P4HB, DNAJB2, MAN1B1, UFD1, PDIA4, HYOU1, SEC61A1, UBE2G2, UBQLN1, SSR1, PREB, CKAP4, ERP29, UBE4B, YOD1, DNAJA2 |
| Cytokine-cytokine receptor interaction | 2.3e-08 | 6.1e-07 | 1.98 | 203 | CXCL8, CCL20, TNFRSF9, CXCL1, VEGFA, BMP2, CXCL10, CXCL2, TNFRSF12A, CXCL3, INHBA, CCL4, LIF, TNF, TNFRSF10B, IL1A, IL6ST, INHBE, IL10RA, IFNLR1, HGF, TNFRSF10A, CSF1, TNFRSF8, IL11, OSMR, CCL2, EGFR, TGFB2, IL7R, FLT1, CXCL11, IL18RAP, FLT4, CCR7, IL12A, FAS, CD70, PDGFB, TNFRSF10C, ACVR2B, CXCR2, EDA2R, TGFBR2, KITLG, CNTF, IL1B, IFNGR2, PRLR, TNFSF9, ACVR1B, CCR4, CD40, IL12B, IL1R2, XCL1, OSM, FLT3LG, PDGFRB, CTF1, GDF5, IL17D, IL4R, CXCR4, IL1RAP, IFNAR1, IL1R1, IL18R1, CSF2RB, CCL8, IL12RB2, VEGFC, NGFR, CX3CL1, CXCL6, RELT, IL20RA, LTA |
| Endocytosis | 9.1e-08 | 2.1e-06 | -1.803 | 241 | PARD3, HSPA1B, AGAP1, HSPA8, RAB7A, SH3KBP1, SNX1, RHOA, CDC42, CHMP5, ARPC2, SNF8, SMAP1, RAB11A, RAB8A, CHMP2A, RAB5B, CAPZB, RAB5C, AP2B1, HRAS, ACAP1, MDM2, SRC, VPS29, UBB, AP2M1, CHMP2B, ARF1, SNX12, VPS28, PIP5K1C, VPS25, ASAP3, RAB11B, IGF1R, ASAP1, CLTB, SNX3, CHMP4B, ARFGAP3, RAB22A, SNX2, FOLR1, CAPZA2, PARD6A, GRK5, WASHC5, VTA1, WASHC2C, ARPC3, KIF5B, RABEP1, CHMP3, WAS, WASHC3, CLTA, ARRB2, CLTC, SNX32, HLA-A, CAV2, PSD3, SNX6, WIPF2, RAB4A, WASHC1, ARF3, SMAD2, SNX4, ARPC1B, STAM, EPS15, PLD1, VPS35, PDCD6IP, TSG101, CHMP6, ARF5, CYTH2, ARPC1A, RUFY1, EPN3, RAB31, EHD3, HLA-B, EPS15L1, IQSEC3, CBLC, LDLR, VPS26B, MVB12A, ARAP1 |
| Retrograde endocannabinoid signaling | 9.3e-08 | 2.1e-06 | -2.037 | 129 | NDUFB4, GRIA4, NDUFAB1, NDUFA9, CACNA1F, NDUFS5, NDUFB10, GNB1, GNAI2, NDUFB3, PLCB1, NDUFA12, GNAQ, PRKCA, NDUFA3, MGLL, NDUFA4, NDUFB5, GNG7, GNAI3, GNG5, NDUFB9, NDUFS4, NDUFS6, NDUFB2, ADCY6, MAPK11, NDUFA2, PRKACB, GNGT1, NDUFA8, NDUFB6, NDUFC1, NDUFB11, NDUFV1, NDUFB1, NDUFS8, NDUFA1, NDUFA5, CACNA1S, ADCY3, CACNA1B, NDUFA6, GNG13, NDUFA10, GABRB3, GNG12, GNAI1, GNB2 |
| Osteoclast differentiation | 1.1e-07 | 2.3e-06 | 2.061 | 117 | NFKBIA, RELB, JUN, NFKB2, JUNB, NFKB1, FOSL1, FOSL2, SQSTM1, RELA, NFATC1, MAP3K14, TNF, CYLD, |

| | | | | | |
|---|---|---|---|---|---|
| | | | | | IL1A, FHL2, JUND, MITF, FOS, NFATC2, CSF1, CHUK, FOSB, TGFB2, BTK, SOCS1, CREB1, FCGR2B, TRAF6, PIK3R1, STAT1, TGFBR2, LCK, GAB2, PIK3CD, IL1B |
| Proteasome | 1.6e-07 | 3.2e-06 | -2.249 | 43 | PSME2, PSMF1, PSMD1, PSMB1, PSMA5, POMP, PSMB2, PSMD14, PSMA3, PSMD4, PSMB6, PSMC1, PSMB3, PSMA7, PSMD7, PSMD8, PSMD12, PSMA4, SEM1, PSMC6, PSMC3, PSME1, PSMB7, PSMD6, PSMD11, PSMB5, PSME4, PSMC2 |
| Kaposi's sarcoma-associated herpesvirus infection | 4.2e-07 | 7.8e-06 | 1.939 | 166 | CXCL8, NFKBIA, JUN, CXCL1, GABARAPL1, VEGFA, CXCL2, NFKB1, RCAN1, CXCL3, ICAM1, MICB, RELA, TICAM1, NFATC1, TP53, CASP3, HLA-E, E2F1, E2F2, IL6ST, IRF7, FOS, EP300, NFATC2, JAK2, MYC, CHUK, GNB5, EIF2AK2, RAF1, CCND1, NFATC3, CREB1, ZFP36, FAS, PDGFB, PIK3R1, STAT1 |
| Vibrio cholerae infection | 1.7e-06 | 3.0e-05 | -2.167 | 48 | ATP6V0E2, KCNQ1, KDELR1, ATP6V1H, SEC61G, ATP6V1E1, ATP6V0E1, GNAS, ERO1A, PRKCA, ARF1, PLCG2, SEC61B, TCIRG1, PRKACB, CFTR, ATP6V0A4, PDIA4, ACTB, SEC61A1, ATP6V0D1, ATP6V1G1, ADCY3 |
| Toll-like receptor signaling pathway | 2.1e-06 | 3.4e-05 | 2.069 | 83 | CXCL8, NFKBIA, JUN, CXCL10, NFKB1, RELA, TICAM1, CCL4, TNF, MAP3K8, IRF7, FOS, CHUK, CXCL11, TIRAP, TLR5, IL12A, TRAF6, PIK3R1, STAT1, TRAF3 |
| RIG-I-like receptor signaling pathway | 2.5e-06 | 4.0e-05 | 2.157 | 55 | CXCL8, NFKBIA, CXCL10, NFKB1, RELA, TNF, CYLD, MAP3K1, IRF7, CHUK, IFIH1, NLRX1, ATG12, IL12A, CASP10, TRAF6, TRAF3, AZI2, NFKBIB, RNF125, RIPK1, IL12B |
| Rheumatoid arthritis | 3.7e-06 | 5.5e-05 | 2.025 | 76 | CXCL8, CCL20, JUN, CXCL1, VEGFA, ICAM1, TNF, IL1A, HLA-DRB5, FOS, CSF1, IL11, CCL2, TGFB2, HLA-DMA, FLT1 |
| Malaria | 4.3e-06 | 6.1e-05 | 2.131 | 36 | CXCL8, ICAM1, SELE, TNF, THBS1, THBS2, HGF, CCL2, TGFB2, HBA2, IL12A, VCAM1, IL1B, CD40, HBA1 |
| Hepatitis B | 5.9e-06 | 7.7e-05 | 1.927 | 128 | CXCL8, NFKBIA, JUN, CREB5, NFKB1, STAT4, RELA, TICAM1, NFATC1, TP53, TNF, CASP3, E2F1, E2F2, MAP3K1, IRF7, SMAD3, BCL2, FOS, EP300, NFATC2, MYC, CHUK, TGFB2, IFIH1, TIRAP, RAF1, CCND1, NFATC3, CREB1, EGR2, EGR3, FAS, STAT5A, CASP10, PIK3R1, STAT1 |
| Herpes simplex infection | 5.7e-06 | 7.7e-05 | 1.845 | 166 | NFKBIA, JUN, NFKB1, PER2, RELA, TICAM1, TP53, TNF, CASP3, HLA-E, SRSF6, PER1, NXF1, IRF7, TAF4B, HLA-DRB5, SRSF7, NXF3, TRAF1, FOS, OAS3, EP300, JAK2, TAF5L, GTF2IRD1, HCFC2, CHUK, SRSF5, CCL2, EIF2AK2, POLR2A, HLA-DMA, IFIH1, TAP1, IL12A, FAS, TRAF6, SRSF1, OAS2, STAT1, TRAF3, TAF13, SRPK1, PTPN11, EIF2AK3, HCFC1, IL1B, NFKBIB, IFNGR2 |
| Legionellosis | 7.2e-06 | 9.0e-05 | 2.095 | 50 | CXCL8, NFKBIA, CXCL1, NFKB2, CXCL2, NFKB1, CXCL3, RELA, HSPA1L, TNF, CASP3 |
| Measles | 9.0e-06 | 1.1e-04 | 1.907 | 113 | TNFAIP3, NFKBIA, NFKB1, RELA, TP53, HSPA1L, TNFRSF10B, CCND2, BBC3, IL1A, IRF7, OAS3, TNFRSF10A, JAK2, CHUK, RAB9A, EIF2AK2, IFIH1, RCHY1, CCND1, CD3G, FCGR2B, IL12A, FAS, STAT5A, TNFRSF10C, TRAF6, PIK3R1, OAS2, STAT1, CCND3, PIK3CD, EIF2AK3, CDK6, IL1B, NFKBIB, ADAR, IFNGR2 |
| Influenza A | 1.1e-05 | 1.2e-04 | 1.843 | 150 | CXCL8, NFKBIA, JUN, CXCL10, NFKB1, ICAM1, RELA, TICAM1, HSPA1L, DDX39B, TNF, TNFRSF10B, NXF1, IL1A, IRF7, HLA-DRB5, NXF3, OAS3, NXT1, EP300, TNFRSF10A, JAK2, BCL2L2-PABPN1, CCL2, EIF2AK2, HLA-DMA, IVNS1ABP, IFIH1, RAF1, NLRX1, IL12A, FAS, CPSF4, TNFRSF10C, PIK3R1, OAS2, STAT1 |
| HTLV-I infection | 1.4e-05 | 1.6e-04 | 1.686 | 245 | ATF3, NFKBIA, RELB, JUN, NFKB2, EGR1, NFKB1, ETS1, POLD3, FOSL1, ICAM1, CDKN2B, RELA, NFATC1, MAP3K14, TP53, ETS2, TNF, HLA-E, E2F1, MSX2, CCND2, E2F2, MAP3K1, SMAD3, HLA-DRB5, TERT, FOS, CHEK1, EP300, NFATC2, POLE3, MYC, CHUK, POLE, WNT11, TGFB2, HLA-DMA, ATR, APC2, SRF, CCND1, NFATC3, CD3G, CREB1, POLE2, EGR2, ZFP36, CREM, FZD8, PDGFB, STAT5A, WNT2B, PIK3R1 |

| | | | | | |
|---|---|---|---|---|---|
| Huntington's disease | 2.2e-05 | 2.4e-04 | -1.743 | 169 | NDUFB4, DNAH3, NDUFAB1, COX7A2, NDUFA9, UQCR11, NDUFS5, NDUFB10, COX7B, UQCRQ, SOD1, NDUFB3, PLCB1, AP2B1, NDUFA12, GNAQ, NDUFA3, COX5A, COX6B1, COX6C, NDUFA4, AP2M1, NDUFB5, HDAC2, COX4I1, NDUFB9, NDUFS4, UQCR10, NDUFS6, CLTB, DCTN2, REST, BAX, NDUFB2, VDAC1, POLR2J, NDUFA2, PPARG, POLR2E, COX7C, POLR2G, COX5B, HDAC1, SDHC, UQCRH, UQCRC2, CLTA, NDUFA8, CLTC, NDUFB6, NDUFC1, SDHB, VDAC3, NDUFB11, NDUFV1, NDUFB1, CREB3L4, COX6A1, NDUFS8, DNAI1, NDUFA1, POLR2L, UQCRC1, NDUFA5, COX8A, DNAH5, NDUFA6, NDUFA10, VDAC2, POLR2K, SLC25A5, COX7A2L, IFT57 |
| DNA replication | 3.2e-05 | 3.4e-04 | 2.04 | 36 | POLD3, MCM4, RFC3, MCM7, RFC5, RNASEH1, MCM3, MCM2, LIG1, RFC2, POLE3, DNA2, POLE, MCM5, POLE2 |
| Dopaminergic synapse | 6.7e-05 | 6.7e-04 | -1.781 | 123 | CALM2, PPP2R5C, PPP3CB, PPP2R5E, GRIA4, GSK3B, PPP3CA, AKT3, GNB1, GNAI2, MAOB, GNAS, PPP2R5D, CAMK2D, PLCB1, GNAQ, PRKCA, ATF2, CALM3, GNG7, GNAI3, GNG5, PPP2R1A, PPP2R2B, PPP1CB, MAPK11, PRKACB, GNGT1, PPP1CA, COMT, KIF5B, PPP2CA, ARRB2, MAOA, CREB3L4, CACNA1B, PPP2R2C, GNAL, GNG13, GSK3A, GNG12, GNAI1, LRTOMT, GNB2, PPP2R5A, PPP1CC, PPP2R3A, PPP2R2D, PLCB3, ITPR2, PRKCG, PLCB4 |
| Leishmaniasis | 6.8e-05 | 6.7e-04 | 1.986 | 65 | NFKBIA, JUN, NFKB1, RELA, TNF, IL1A, HLA-DRB5, FOS, JAK2, TGFB2, HLA-DMA, IL12A, TRAF6, STAT1, NOS2, IL1B, NFKBIB, IFNGR2, IL12B |
| Small cell lung cancer | 7.5e-05 | 7.1e-04 | 1.857 | 92 | NFKBIA, GADD45A, GADD45B, NFKB1, BIRC3, CDKN2B, RELA, BIRC2, TP53, CASP3, E2F1, E2F2, BCL2, TRAF4, TRAF1, MYC, CHUK, POLK, ITGAV, CCND1, GADD45G, TRAF6, PIK3R1, TRAF3, ZBTB17, NOS2, PIK3CD, ITGA6, CDK6 |
| Vascular smooth muscle contraction | 7.8e-05 | 7.1e-04 | -1.756 | 107 | PRKG1, CALM2, RHOA, CACNA1F, GNAS, PLCB1, GNAQ, PRKCA, CALM3, MYL6B, AGTR1, MYLK2, MYL6, MAP2K2, PLA2G6, PPP1CB, ACTA2, RAMP1, RAMP2, ADCY6, PRKACB, PPP1CA, MYL9, PRKCE, EDNRA, ARAF, CACNA1S, ADCY3, KCNMA1, PLA2G12A, PPP1CC, PPP1R12B, PLCB3, ITPR2, MRVI1, PRKCG, PLCB4, PLA2G4A |
| Inflammatory bowel disease (IBD) | 9.5e-05 | 8.2e-04 | 1.952 | 56 | JUN, NFKB1, STAT4, RELA, NFATC1, TNF, IL1A, SMAD3, HLA-DRB5, FOXP3, TGFB2, HLA-DMA, IL18RAP, TLR5, IL12A, TBX21, STAT1 |
| Th1 and Th2 cell differentiation | 9.1e-05 | 8.2e-04 | 1.857 | 86 | NFKBIA, JUN, NFKB1, STAT4, RELA, NFATC1, NFKBIE, HLA-DRB5, FOS, NFATC2, JAK2, CHUK, HLA-DMA, NFATC3, NOTCH1, CD3G, IL12A, DLL4, TBX21, ZAP70, STAT5A, JAG1, STAT1, LCK, CD4, NFKBIB, IFNGR2, MAML1 |
| Th17 cell differentiation | 9.7e-05 | 8.2e-04 | 1.821 | 99 | NFKBIA, JUN, NFKB1, RELA, NFATC1, NFKBIE, IL6ST, SMAD3, RUNX1, HLA-DRB5, FOS, AHR, NFATC2, JAK2, CHUK, IL27RA, FOXP3, HLA-DMA, NFATC3, CD3G, RARA, TBX21, ZAP70, STAT5A, STAT1, IRF4, TGFBR2, LCK, CD4, IL1B, NFKBIB, IFNGR2 |
| AGE-RAGE signaling pathway in diabetic complications | 1.3e-04 | 0.001 | 1.862 | 98 | CXCL8, JUN, VEGFA, EGR1, NFKB1, ICAM1, PIM1, RELA, NFATC1, SELE, F3, TNF, CASP3, IL1A, SMAD3, BCL2, JAK2, CCL2, TGFB2, CCND1, STAT5A, PIK3R1, STAT1, VCAM1, TGFBR2 |
| Apoptosis | 1.3e-04 | 0.001 | 1.744 | 130 | NFKBIA, JUN, GADD45A, GADD45B, NFKB1, PMAIP1, BIRC3, RELA, BIRC2, MAP3K14, TP53, MCL1, TNF, CASP3, TNFRSF10B, BBC3, HRK, BCL2, CASP2, TRAF1, FOS, TNFRSF10A, CHUK, BCL2L11, RAF1, FAS, NTRK1, GADD45G, TNFRSF10C, CASP10, PIK3R1 |
| Bladder cancer | 1.4e-04 | 0.001 | 1.981 | 40 | CXCL8, VEGFA, RASSF1, TP53, E2F1, E2F2, THBS1, MYC, TYMP, EGFR, HBEGF, RAF1, CCND1 |

| | | | | | |
|---|---|---|---|---|---|
| Cytosolic DNA-sensing pathway | 1.3e-04 | 0.001 | 1.977 | 43 | NFKBIA, CXCL10, NFKB1, RELA, CCL4, IRF7, CGAS, POLR3A, CHUK, POLR3D, POLR3F, CASP1, POLR1C, POLR3C, POLR2H, IL1B, NFKBIB, ADAR, RIPK1, IKBKB, POLR3K |
| MAPK signaling pathway | 1.3e-04 | 0.001 | 1.592 | 286 | RELB, JUN, GADD45A, DUSP1, VEGFA, GADD45B, NFKB2, NFKB1, DUSP3, EPHA2, DUSP16, EFNA1, RELA, DUSP8, NFATC1, MAP3K14, TP53, HSPA1L, TNF, CASP3, DUSP10, CACNB1, DUSP6, DUSP5, CACNA1E, MAP3K8, RASGRP2, IL1A, MAP3K1, CACNB4, JUND, FOS, CACNG7, HGF, CSF1, MYC, CHUK, CRKL, EGFR, TGFB2, FLT1, FLT4, DUSP4, RAF1, SRF, PLA2G4D, NFATC3, FGF17, FAS, MKNK2, NTRK1, PDGFB, GADD45G, TRAF6 |
| Pathways in cancer | 1.5e-04 | 0.001 | 1.449 | 495 | CXCL8, NFKBIA, JUN, GADD45A, VEGFA, BMP2, GADD45B, NFKB2, NFKB1, PMAIP1, ETS1, BIRC3, PIM1, MSH6, CDKN2B, STAT4, RASSF1, RELA, BIRC2, TP53, CASP3, E2F1, CCND2, CAMK2A, NKX3-1, BBC3, E2F2, RASGRP2, BRCA2, IL6ST, RPS6KB1, ABL1, SMAD3, RUNX1, TERT, EGLN2, BCL2, TRAF4, MITF, TRAF1, FOS, NFE2L2, EP300, HGF, JAK2, MYC, CHUK, POLK, GNB5, CRKL, WNT11, ITGAV, EGFR, TGFB2, CCDC6, IL7R, SP1, BCL2L11, APC2, FLT4, TCF7L1, RAF1, CCND1, MSH2, FGF17, NOTCH1, IL12A, DLL4, FAS, RARA, FRAT2, FZD8, LRP6, PTGER2, NTRK1, PDGFB, BDKRB2, DAPK2, STAT5A, GADD45G, ARHGEF1, TRAF6, JAG1, WNT2B, PIK3R1, STAT1, FGFR2, TRAF3, NCOA3, ZBTB17, NOS2, TGFBR2, KITLG, CDH1, ARNT, CCND3, GSTA1, CBL, TCF7, PIK3CD, FZD5, AXIN1, ITGA6, CDK6, RASGRP1 |
| Transcriptional misregulation in cancer | 2.1e-04 | 0.001 | 1.647 | 159 | CXCL8, GADD45A, PLAU, GADD45B, NFKB1, PER2, BIRC3, RELA, PAX3, TP53, CEBPB, NFKBIZ, DDX5, CCND2, DUSP6, CCNT1, ETV1, RUNX1, MITF, TRAF1, LDB1, RUNX2, SIX4, MYC, CCNT2, BCL6, POLK, FLT1, SP1, KLF3, IGFBP3, REL, RARA, KMT2A, NTRK1, GADD45G |
| Cardiac muscle contraction | 2.5e-04 | 0.002 | -1.798 | 68 | ATP1B3, COX7A2, CACNA1F, UQCR11, COX7B, UQCRQ, CACNA2D3, COX5A, COX6B1, COX6C, TPM3, COX4I1, UQCR10, TPM2, MYL4, COX7C, CACNA2D1, COX5B, UQCRH, UQCRC2, RYR2, COX6A1, CACNG6, ATP1B2, TNNI3, UQCRC1, COX8A, CACNA1S, COX7A2L, FXYD2, UQCRB, UQCRHL, UQCRFS1, CYC1, COX6B2 |
| Chronic myeloid leukemia | 3.1e-04 | 0.002 | 1.766 | 78 | NFKBIA, GADD45A, GADD45B, NFKB1, RELA, TP53, E2F1, E2F2, ABL1, SMAD3, RUNX1, MYC, CHUK, POLK, CRKL, SHC3, TGFB2, RAF1, CCND1, STAT5A, GADD45G, PIK3R1, TGFBR2, PTPN11, CBL, GAB2, PIK3CD, CDK6 |
| Epithelial cell signaling in Helicobacter pylori infection | 2.9e-04 | 0.002 | 1.834 | 67 | CXCL8, NFKBIA, JUN, CXCL1, NFKB1, RELA, MAP3K14, CASP3, CHUK, EGFR, HBEGF, ATP6V1E2, CXCR2, ATP6V0C, GIT1 |
| Chagas disease (American trypanosomiasis) | 3.9e-04 | 0.003 | 1.743 | 92 | CXCL8, NFKBIA, JUN, NFKB1, RELA, TICAM1, TNF, SMAD3, FOS, CHUK, CCL2, TGFB2, CD3G, IL12A, FAS, BDKRB2, TRAF6, PIK3R1, NOS2, TGFBR2 |
| Gastric acid secretion | 4.9e-04 | 0.003 | -1.757 | 68 | CALM2, ATP1B3, KCNQ1, GNAI2, GNAS, CAMK2D, PLCB1, GNAQ, PRKCA, CA2, CALM3, GNAI3, MYLK2, ADCY6, PRKACB, EZR, CFTR, ACTB, ATP1B2, ADCY3, GNAI1, PLCB3, ITPR2, PRKCG, PLCB4, KCNJ10, SLC26A7, CALML6, CAMK2B, GAST |
| Gap junction | 7.6e-04 | 0.005 | -1.684 | 80 | PRKG1, CDK1, TUBB4B, TUBB, TUBA1C, TUBA1B, MAP2K5, GNAI2, GNAS, PLCB1, GNAQ, PRKCA, HRAS, GRB2, SRC, GNAI3, SOS2, MAP2K2, ADCY6, PRKACB, TUBA1A |
| Lysosome | 8.9e-04 | 0.005 | -1.586 | 121 | AP3S1, CTSL, ATP6V1H, CTSD, CD63, M6PR, TPP1, GNS, CTSB, AP3B1, HEXB, DNASE2, SORT1, CLTB, LAPTM4A, ARSB, AP3D1, TCIRG1, CTSV, CTSZ, PPT1, LITAF, CLTA, LAMP1, CLTC, GM2A, HYAL1, ARSA, ATP6V0A4, |

| | | | | | |
|---|---|---|---|---|---|
| | | | | | ATP6V0D1, SUMF1, CLN3, LAMP2, NAGPA, LAPTM4B, ABCB9, HEXA, GGA1, GUSB, CTSC |
| Mismatch repair | 8.8e-04 | 0.005 | 1.865 | 23 | EXO1, POLD3, MSH6, RFC3, RFC5, LIG1, RFC2, MLH3, MSH2 |
| Adrenergic signaling in cardiomyocytes | 0.001 | 0.006 | -1.588 | 136 | CALM2, PPP2R5C, PPP2R5E, ATP1B3, KCNQ1, AKT3, CACNA1F, GNAI2, GNAS, PPP2R5D, CAMK2D, PLCB1, CACNA2D3, GNAQ, PRKCA, ATF2, TPM3, CALM3, AGTR1, GNAI3, PPP2R1A, PPP2R2B, TPM2, PPP1CB, MYL4, ADCY6, MAPK11, PRKACB, CACNA2D1, PPP1CA, RAPGEF4, PPP2CA, PPP1R1A, RYR2, CREB3L4, CACNG6, ATP1B2, TNNI3, CACNA1S, ADCY3, PPP2R2C |
| Cell cycle | 0.001 | 0.006 | -1.586 | 124 | CCNB1, CDC20, PLK1, BUB1, TTK, CCNA2, CDKN2C, CCNB2, CDK1, PTTG1, YWHAE, CDC25C, YWHAB, YWHAZ, GSK3B, CDC25B, ORC4, BUB3, CUL1, BUB1B, DBF4, TFDP2, ANAPC5, STAG1, MDM2, RAD21, CCNA1, ANAPC11, ANAPC10, HDAC2, SMC1B, RBX1, MAD2L2, HDAC1, YWHAQ, MAD2L1, SKP1, STAG2, SFN, CDC26, ESPL1, CDC16, CDKN1B, CDKN1C, CDC27, SMAD2 |
| Glycosaminoglycan biosynthesis - heparan sulfate / heparin | 9.8e-04 | 0.006 | -1.867 | 23 | EXTL2, HS6ST2, EXT2, HS2ST1, HS6ST1, EXTL3, EXT1, HS3ST1, GLCE, B3GAT3, B4GALT7, NDST3, B3GALT6 |
| MicroRNAs in cancer | 9.5e-04 | 0.006 | 1.543 | 165 | PLAU, VEGFA, NFKB1, SLC7A1, PIM1, RASSF1, CDC25A, DNMT1, TP53, MCL1, CASP3, E2F1, CCND2, CDCA5, E2F2, ABL1, GLS, THBS1, BCL2, EP300, MYC, CRKL, SIRT1, EGFR, TGFB2, SOX4, BCL2L11, APC2, MIRLET7D, RAF1, SPRY2, IRS1, SOCS1, CCND1, NOTCH1, TNXB |
| Pancreatic cancer | 0.001 | 0.006 | 1.684 | 75 | GADD45A, VEGFA, GADD45B, NFKB1, RELA, TP53, E2F1, E2F2, BRCA2, RPS6KB1, SMAD3, CHUK, POLK, EGFR, TGFB2, RAF1, CCND1, GADD45G, PIK3R1, STAT1, TGFBR2 |
| Renin secretion | 0.001 | 0.006 | -1.732 | 56 | CALM2, PPP3CB, PPP3CA, CACNA1F, GNAI2, GNAS, PLCB1, GNAQ, CTSB, CALM3, AGTR1, GNAI3, ADORA1, ADCY6, PRKACB, PDE3B, EDNRA, PDE1C, CACNA1S, KCNMA1, GNAI1, PLCB3, ADCYAP1R1, ITPR2, PLCB4 |
| Salmonella infection | 0.001 | 0.006 | 1.719 | 77 | CXCL8, JUN, CXCL1, CXCL2, NFKB1, CXCL3, RELA, CCL4, IL1A, FOS, KLC2, PKN3 |
| Bacterial invasion of epithelial cells | 0.001 | 0.007 | -1.66 | 67 | PTK2, RHOA, CDC42, DOCK1, ARPC2, ELMO1, CTNNA1, PIK3CB, RAC1, ARHGAP10, SRC, CLTB, PXN, MAD2L2, ARPC3, WAS, CLTA, CLTC, CAV2, WASF1, RHOG, ARPC1B, ACTB, CTNNB1, CTNNA3, ARPC1A, CBLC, SHC1, CD2AP, ARPC5 |
| Fanconi anemia pathway | 0.001 | 0.007 | 1.745 | 52 | PALB2, BRCA2, USP1, TOP3A, ERCC4, POLK, ATR, BRIP1, FANCB, EME2, FANCA, BLM, EME1, FAN1, TOP3B, FANCE, TELO2, POLN, BRCA1, FANCM, ATRIP, RMI2, REV1 |
| Propanoate metabolism | 0.001 | 0.007 | -1.788 | 31 | PCCA, LDHB, LDHC, BCKDHA, SUCLA2, EHHADH, SUCLG1, LDHA, ECHS1, ACACA, HADHA, ALDH6A1, SUCLG2, MCEE, DLD, HIBCH, ACAT1, PCCB |
| Regulation of actin cytoskeleton | 0.001 | 0.007 | -1.486 | 203 | PTK2, DIAPH3, TIAM1, RHOA, CFL1, CDC42, DOCK1, DIAPH2, FGF13, ARPC2, MYH10, VAV3, PIK3CB, RAC1, PFN2, HRAS, SRC, PIP5K1C, IQGAP2, BRK1, MYL12B, SOS2, ITGB4, MYLK2, MYH14, FGF1, MAP2K2, PPP1CB, CYFIP2, PXN, PPP1CA, EZR, ARPC3, RAC2, MYL9, SCIN, FGF22, WAS, PFN1, LIMK1, FGF18, PIP4K2A, PAK2, WASF1, ARPC1B, ACTB, VAV2, ARAF, ARHGAP35, ITGB5, ABI2, FGF9, ITGAL, FGD3, ARPC1A, ITGAE, SSH3, FGF23, GNG12, FGF20, PDGFC, PDGFA, MSN, ARPC5, PPP1CC, RRAS2, PPP1R12B, PAK5, ACTN1, FGF21, RRAS |
| Ubiquitin mediated proteolysis | 0.001 | 0.007 | -1.567 | 133 | CDC20, UBE2C, UBE2S, UBE2L3, MID1, UBE2N, UBE2E3, UBE2D2, CUL1, UBE2E2, UBE2L6, PIAS1, ANAPC5, MDM2, UBE2B, UBE2F, ANAPC11, ANAPC10, KLHL13, STUB1, UBE2E1, UBE2K, CDC34, PPIL2, ELOB, RBX1, FBXO2, UBE2G1, UBE2H, SKP1, PRKN, UBE2D4, CDC26, |

| | | | | | |
|---|---|---|---|---|---|
| | | | | | DDB2, BTRC, CDC16, CDC27, TRIP12, UBE2G2, VHL, CUL4B, UBA6, UBE2Q2, UBOX5, UBE4B |
| Pancreatic secretion | 0.002 | 0.008 | -1.631 | 83 | ATP1B3, RHOA, KCNQ1, RAB11A, RAB8A, GNAS, PLCB1, RAC1, GNAQ, PRKCA, ATP2A1, RAB3D, CA2, ADCY6, SLC4A4, RAP1A, CFTR, RYR2, CELA2B, CEL, ATP1B2, ADCY3, KCNMA1, FXYD2, PLA2G12A |
| Cellular senescence | 0.002 | 0.009 | 1.554 | 152 | CXCL8, GADD45A, GADD45B, NFKB1, ZFP36L1, ETS1, CDKN2B, SQSTM1, RELA, NFATC1, CDC25A, TP53, HLA-E, E2F1, CCND2, E2F2, IL1A, SMAD3, RAD9A, CHEK1, TRAF3IP2, NFATC2, ZFP36L2, MYC, SIRT1, LIN54, TSC2, TGFB2, ATR, RAF1, CCND1, HIPK1, NFATC3, IGFBP3 |
| Colorectal cancer | 0.002 | 0.009 | 1.688 | 71 | JUN, GADD45A, GADD45B, PMAIP1, MSH6, TP53, CASP3, BBC3, SMAD3, BCL2, FOS, MYC, POLK, TGFB2, BCL2L11, APC2, TCF7L1, RAF1, CCND1, MSH2, GADD45G, PIK3R1, TGFBR2, TCF7, PIK3CD, AXIN1 |
| Hepatitis C | 0.002 | 0.01 | 1.595 | 113 | CXCL8, NFKBIA, NFKB1, CLDN1, RELA, TICAM1, TP53, TNF, IRF1, IRF7, OAS3, CHUK, EGFR, EIF2AK2, RAF1, TRAF6, PIK3R1, OAS2, STAT1, PSME3, TRAF3 |

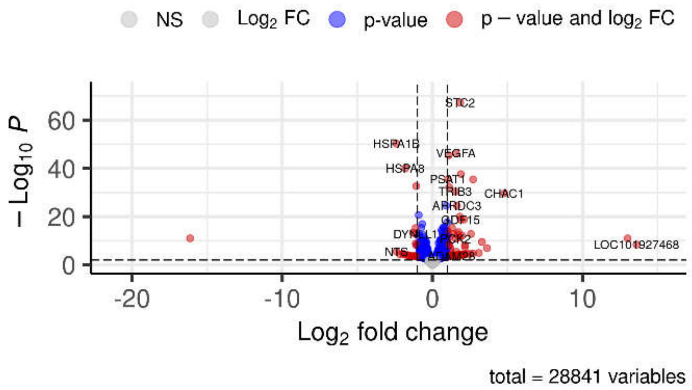


**A**

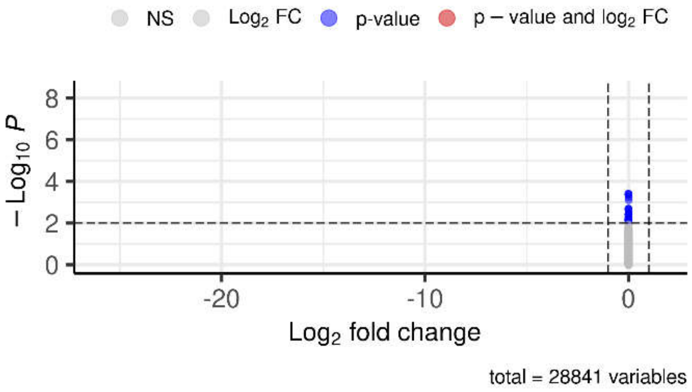


**B**

**Figure S4.** OL9-119 effect on A) WT HEK293A cells treated with 5 µM OL9-119 vs untreated cells; B) PARP1-KO HEK293A cells treated with 5 µM OL9-119 vs cells treated with 0.1% DMSO. Volcano Plots for differential gene expression under the influence of OL9-119.

**Table S3.** Gene set enrichment analysis results of OL9-119 effect on gene expression: pathways significantly associated with differentially expressed genes upon treatment with OL9-119 or DMSO (WT HEK293A cells treated with 5 μM OL9-119 vs cells treated with 0.1% DMSO).

| Pathway | *p*-Value | *p*-adj | NES | Number | Genes |
|---|---|---|---|---|---|
| Ribosome | 2.4e-33 | 7.4e-31 | -3.092 | 129 | RPS27L, MRPL33, MRPL23, RPL35, RPL7A, RPL24, RPL36AL, MRPL13, RPL35A, RPL22, UBA52, RPS26, RPL38, RPS4X, RPL29, RPL6, MRPL18, RPL19, RPS25, RPLP0, RPS3A, RPL26L1, RPS7, RPL34, MRPL36, MRPL20, RPL23A, FAU, MRPL11, RPS27A, RPL23, RPL5, RPS24, RPS12, RPL41, RPL27, RPL11, MRPL21, RPS13, RPS6, MRPL14, RPS14, RPL36, RPS8, MRPS9, RPL7, MRPS11, RPS16, MRPL35, RPL21, RPL27A, RPS5, RPL14, RPL9, MRPL27, RPL37A, MRPS7, RPS18, RPLP1, MRPL28, MRPS21, RPL10, RPL10A, RPL4, RPSA, MRPL1, RPS27, MRPS14, RPL18, RPL12, RPS20, MRPS18C, MRPL30, MRPS6, RPL37, MRPL24, RPS23, RPS19, RPLP2, RPL13, MRPS15, RPS2, RPL31, MRPL34, RPL15, RPL3, RPL8, RPL39, RPS11, MRPL3, MRPL9, MRPS12, RPL13A, RPL28, RPS15, RPS3, MRPL15, RPL36A, RPS9, MRPS16, MRPL12 |
| Oxidative phosphorylation | 5.0e-21 | 7.9e-19 | -2.852 | 98 | NDUFB4, COX7A2, COX7B, NDUFB3, NDUFA4, ATP6V0E1, NDUFS5, NDUFA2, UQCRQ, ATP6V1E1, NDUFA12, COX7A2L, NDUFAB1, NDUFB9, ATP6V0E2, NDUFA5, UQCR10, UQCR11, COX17, NDUFA3, NDUFA8, COX6C, NDUFS6, NDUFA6, SDHD, COX5A, COX6B1, NDUFB2, NDUFB6, UQCRH, NDUFC1, COX7C, ATP6V0A4, NDUFB10, NDUFS4, NDUFB5, COX4I1, COX8A, SDHC, PPA1, UQCRB, NDUFA1, UQCRC2, NDUFA9, PPA2, COX5B, NDUFS3, ATP6V0D1, NDUFB1, ATP6V1G1, UQCRFS1, UQCRC1, NDUFC2, NDUFB11, ATP6V1H, NDUFS8, SDHA, ATP6V0B, NDUFS7, ATP6V0A1 |
| Parkinson's disease | 2.5e-20 | 2.7e-18 | -2.794 | 109 | NDUFB4, COX7A2, UBE2L3, COX7B, NDUFB3, NDUFA4, NDUFS5, NDUFA2, PARK7, UQCRQ, VDAC3, NDUFA12, COX7A2L, UBB, VDAC1, GNAI2, NDUFAB1, NDUFB9, CYCS, SNCA, NDUFA5, UQCR10, UQCR11, NDUFA3, NDUFA8, COX6C, NDUFS6, NDUFA6, SDHD, COX5A, UBE2G2, UCHL1, COX6B1, NDUFB2, NDUFB6, UQCRH, NDUFC1, COX7C, NDUFB10, NDUFS4, NDUFB5, COX4I1, SLC25A5, COX8A, SDHC, UQCRB, NDUFA1, SLC25A4, UQCRC2, NDUFA9, COX5B, NDUFS3, GNAI3, NDUFB1, GNAI1, UQCRFS1, UQCRC1, NDUFC2, APAF1, NDUFB11, NDUFS8, SDHA, VDAC2, NDUFS7 |
| Alzheimer's disease | 3.9e-18 | 3.0e-16 | -2.586 | 144 | NDUFB4, COX7A2, CALM2, COX7B, NDUFB3, NDUFA4, CALM1, NDUFS5, NDUFA2, UQCRQ, CALM3, NDUFA12, COX7A2L, NDUFAB1, NDUFB9, CYCS, SNCA, HSD17B10, NDUFA5, UQCR10, UQCR11, NDUFA3, CACNA1F, NDUFA8, COX6C, NDUFS6, NDUFA6, SDHD, COX5A, COX6B1, RTN3, NDUFB2, NDUFB6, UQCRH, CDK5, NDUFC1, |

| | | | | | |
|---|---|---|---|---|---|
| | | | | | COX7C, GAPDH, NDUFB10, NDUFS4, NDUFB5, COX4I1, COX8A, SDHC, UQCRB, NDUFA1, PPP3CB, UQCRC2, NDUFA9, COX5B, CASP7, NDUFS3, NDUFB1, PSENEN, UQCRFS1, UQCRC1, NDUFC2, APAF1, ATP2A1, NDUFB11, APBB1, NDUFS8, PPP3CC, MME, SDHA, MAPK3 |
| Spliceosome | 2.0e-17 | 1.3e-15 | -2.637 | 131 | HSPA1B, HSPA8, LSM3, HNRNPM, SRSF3, DDX23, SF3B5, PPIH, SNRPE, SNRPG, PHF5A, SNRPD2, SNRPD3, RBMX, SNRPF, SRSF9, CWC15, SNRPC, SF3B6, HNRNPA1, SRSF2, DDX5, SNRPB2, LSM5, SRSF1, TXNL4A, SNRPD1, NCBP2, SF3B4, PRPF18, LSM7, EFTUD2, PPIL1, PRPF31, SNRPA1, CHERP, RBM25, LSM8, SNU13, SNRNP70, ALYREF, HNRNPC, RBM8A, HNRNPA3, LSM4, SNRNP27, HSPA1A, SRSF8, PRPF38B, ACIN1, BCAS2, ISY1, CTNNBL1, SNRPB, EIF4A3, LSM2, ZMAT2, PCBP1, HNRNPK, SF3B3, DDX46, RBM22 |
| Huntington's disease | 9.9e-14 | 5.1e-12 | -2.35 | 169 | NDUFB4, COX7A2, COX7B, NDUFB3, NDUFA4, POLR2K, NDUFS5, POLR2L, NDUFA2, UQCRQ, VDAC3, NDUFA12, COX7A2L, VDAC1, NDUFAB1, NDUFB9, CYCS, SOD1, NDUFA5, UQCR10, POLR2E, SOD2, UQCR11, NDUFA3, NDUFA8, COX6C, NDUFS6, NDUFA6, SDHD, COX5A, POLR2F, COX6B1, HDAC1, NDUFB2, NDUFB6, UQCRH, AP2M1, NDUFC1, COX7C, NDUFB10, NDUFS4, NDUFB5, POLR2G, COX4I1, SLC25A5, COX8A, SDHC, UQCRB, NDUFA1, CLTA, BAX, SLC25A4, UQCRC2, POLR2C, DCTN2, NDUFA9, POLR2J, TGM2, HDAC2, COX5B, CLTB, NDUFS3, NDUFB1, DNAH5, POLR2I, UQCRFS1, POLR2D, UQCRC1, NDUFC2, APAF1 |
| Proteasome | 1.5e-12 | 6.9e-11 | -2.619 | 43 | POMP, PSMA3, PSMB2, PSMA5, PSMA4, PSMB5, PSME2, PSMD6, PSMB1, PSME3, PSMC3, PSMA7, PSMD1, PSMD12, PSMC2, SEM1, PSMB7, PSMF1, PSME1, PSMD4, PSMD7, PSMB3, PSMC5, PSMD8, PSMC4, PSMB4, PSMB6, PSMD14, PSMC6, ADRM1, PSMB10, PSMC1, PSMD3, PSMB9, PSMD11 |
| Protein processing in endoplasmic reticulum | 4.7e-10 | 1.9e-08 | -2.154 | 157 | HSPA1B, HSPA8, DNAJB1, DNAJA1, HSPH1, HSP90AB1, VCP, SSR2, SEC61G, DNAJA2, DAD1, HSP90AA1, SSR3, RBX1, UFD1, UBE2D2, SSR1, BCAP31, SEC61B, SEC61A1, SAR1A, PDIA3, RPN2, UBE2G2, SKP1, RPN1, YOD1, DERL2, SSR4, SAR1B, CALR, BAG2, UBE2D3, HSP90B1, EIF2S1, BAX, OS9, HSPA1A, SEC62, PDIA6, SEC13, PLAA, PREB, HSPBP1, P4HB, TRAM1, UBE4B, STUB1, STT3A, DNAJB11, FBXO2, BAG1, SEC24A, DNAJB2, ERO1A, GANAB, STT3B, TUSC3, HSPA4L, PRKCSH, UBQLN1, LMAN2 |
| Non-alcoholic fatty liver disease (NAFLD) | 1.9e-09 | 6.6e-08 | -2.15 | 135 | NDUFB4, COX7A2, COX7B, NDUFB3, NDUFA4, CDC42, NDUFS5, NDUFA2, UQCRQ, NDUFA12, COX7A2L, NDUFAB1, NDUFB9, CYCS, NDUFA5, UQCR10, UQCR11, NDUFA3, NDUFA8, COX6C, NDUFS6, NDUFA6, SDHD, COX5A, RAC1, COX6B1, |

| | | | | | |
|---|---|---|---|---|---|
| | | | | | NDUFB2, NDUFB6, UQCRH, NDUFC1, COX7C, NDUFB10, NDUFS4, NDUFB5, COX4I1, COX8A, SDHC, UQCRB, NDUFA1, EIF2S1, BAX, UQCRC2, NDUFA9, ADIPOR1, COX5B, CASP7, GSK3A, NDUFS3, NDUFB1, UQCRFS1, UQCRC1, NDUFC2, IL6, MAP3K11, NDUFB11, CYP2E1, NDUFS8, SDHA, MLXIP |
| Oocyte meiosis | 3.1e-08 | 9.6e-07 | -2.15 | 109 | CALM2, PTTG1, YWHAB, CALM1, CCNB1, YWHAZ, CALM3, RBX1, AURKA, CDK1, YWHAE, YWHAQ, SKP1, BUB1, CCNE2, PPP2CB, YWHAH, PPP1CA, CCNB2, MAD2L1, PPP2CA, CDC20, PPP2R1B, PPP2R5D, CDK2, SMC1B, PPP3CB, CDC26, SPDYA, MAD2L2, ANAPC13, YWHAG, ANAPC11, PPP2R5B, PPP2R1A, PPP2R5C, PPP3CC, ANAPC10, MAPK3 |
| TNF signaling pathway | 2.9e-07 | 8.2e-06 | 2.066 | 106 | CEBPB, NFKBIA, JUN, TNFAIP3, JUNB, CXCL3, CREB1, JAG1, CXCL2, SELE, ATF4, CREB5, ICAM1, PIK3CA, MMP9, TAB2, PTGS2, TRAF3, MAPK9, VEGFC, TRAF1, ITCH, NFKB1, CXCL10, BIRC2, BIRC3, MAP3K7, PIK3R1, SOCS3, MAP3K8, TAB1, MAP2K7, MAPK10, IKBKB, LTA, PIK3CD, TAB3, TRAF5, CHUK, DNM1L, IL18R1, MAPK8 |
| Retrograde endocannabinoid signaling | 3.5e-07 | 9.1e-06 | -1.987 | 129 | NDUFB4, NDUFB3, NDUFA4, NDUFS5, NDUFA2, NDUFA12, GNAI2, NDUFAB1, NDUFB9, NDUFA5, NDUFA3, CACNA1F, GRIA4, NDUFA8, NDUFS6, NDUFA6, NDUFB2, NDUFB6, NDUFC1, MGLL, NDUFB10, NDUFS4, NDUFB5, GNG5, NDUFA1, NDUFA9, NDUFS3, GNAI3, GNG11, MAPK11, NDUFB1, GNAI1, GNGT1, NDUFC2, NDUFB11, PRKCG, NDUFS8, MAPK3, GNG4, NDUFS7, KCNJ3, GNB3, GNG7 |
| Cardiac muscle contraction | 4.3e-07 | 1.0e-05 | -2.178 | 68 | TPM3, COX7A2, COX7B, ATP1B3, UQCRQ, COX7A2L, UQCR10, UQCR11, CACNA1F, COX6C, COX5A, COX6B1, UQCRH, COX7C, COX4I1, COX8A, ATP1B1, UQCRB, TPM4, UQCRC2, TNNI3, COX5B, TPM1, UQCRFS1, UQCRC1 |
| Phagosome | 5.3e-07 | 1.2e-05 | -1.953 | 135 | VAMP3, TUBB, ACTB, ATP6V0E1, SEC61G, TUBB4B, ATP6V1E1, ACTG1, RAB7A, TUBA1C, ATP6V0E2, TUBA1A, SEC61B, SEC61A1, HLA-DRB1, RAB5C, RAC1, TUBA1B, TUBB6, COLEC12, HLA-A, M6PR, HLA-DQB1, ATP6V0A4, C1R, CALR, HLA-DMB, CTSL, PIK3C3, RAB5B, LAMP1, ATP6V0D1, ATP6V1G1, DYNC1I2, CTSS, DYNC1LI2, TUBB8, ATP6V1H, ATP6V0B, HLA-DPA1, ATP6V0A1, ATP6V1A, ATP6V0C, ITGB2, HLA-DQA2, HLA-DQA1, ITGA5, STX12, LAMP2, RILP, SEC22B, DYNC1LI1, ATP6V1B2, HLA-E, NCF2, HLA-DPB1, TUBA8, ATP6V1C1, TUBB4A, CLEC4M, HLA-B, PLA2R1, SCARB1 |
| Antigen processing and presentation | 5.9e-06 | 1.1e-04 | -2.102 | 60 | HSPA1B, HSPA8, HSP90AB1, HSP90AA1, HSPA4, PSME2, B2M, HLA-DRB1, PDIA3, PSME3, KLRC2, HLA-A, RFXANK, PSME1, HLA-DQB1, CALR, HLA-DMB, CTSL, HSPA1A, NFYA, CTSS, TAPBP, IFI30 |

| | | | | | |
|---|---|---|---|---|---|
| Protein export | 5.7e-06 | 1.1e-04 | -2.163 | 23 | SRP9, SEC61G, SEC61B, SEC61A1, SPCS1, SRP14, SRPRB, SPCS2, SEC11C, SPCS3, SEC11A, SEC62, IMMP2L |
| Osteoclast differentiation | 1.7e-05 | 2.9e-04 | 1.858 | 117 | NFKBIA, JUN, GAB2, RELB, JUNB, MITF, CREB1, TEC, FOSL2, IL1A, PIK3CA, TAB2, MAPK9, JUND, NFATC1, FYN, BTK, SQSTM1, NFKB1, FHL2, FOSB, OSCAR, MAP3K7, PIK3R1, LCK, SOCS3, STAT1, TAB1, MAP2K7, MAPK10, CYLD, IKBKB, PIK3CD, CHUK, CSF1R, FCGR2B, MAPK8 |
| Signaling pathways regulating pluripotency of stem cells | 1.8e-05 | 2.9e-04 | 1.826 | 132 | ID4, INHBE, KLF4, ID3, SETDB1, FZD8, AXIN1, PCGF6, PIK3CA, MYC, KAT6A, JAK2, RAF1, IL6ST, STAT3, RIF1, ACVR1, PCGF3, SMAD9, ID1, ZFHX3, SMAD5, DVL1, WNT4, PIK3R1, DLX5, WNT9A, SMAD3, ACVR2A, LEFTY2, PIK3CD, SMAD1, LIFR, KRAS, FZD1, SKIL, PCGF5, LHX5, DVL2, HESX1, PCGF1, ONECUT1, HAND1, FGF2, WNT3, OTX1, FZD7, WNT5B, POU5F1, APC, BMPR2, PAX6, INHBA, FZD4, MAPK14, ZIC3, INHBC, SMAD2, BMPR1A, FGFR2, WNT6 |
| Vibrio cholerae infection | 1.6e-05 | 2.9e-04 | -2.048 | 48 | ACTB, ATP6V0E1, SEC61G, ATP6V1E1, ACTG1, ATP6V0E2, SEC61B, SEC61A1, KDELR1, ATP6V0A4, GNAS, CFTR, ARF1, ATP6V0D1, ATP6V1G1, KDELR2, ATP6V1H, ATP6V0B, ERO1A, ATP6V0A1, ATP6V1A, ATP6V0C, PLCG2, PRKACB, PRKACA |
| Lysosome | 3.0e-05 | 4.6e-04 | -1.799 | 121 | LITAF, GLA, AP3S1, M6PR, CD63, AP1S3, LAPTM4B, ATP6V0A4, CTSV, PPT1, AP1S2, TPP1, CTSD, AP4B1, PPT2, CTSC, CLTA, HEXB, GGA1, CTSL, GM2A, CTSH, CD164, CLN5, LAMP1, CTSA, CLTB, AP1S1, ATP6V0D1, HYAL1, FUCA1, CTSZ, AP1M2, CTSS, PLA2G15, ATP6V1H, ATP6V0B, ACP2, ASAH1, ATP6V0A1, GUSB, CTSB, LIPA, ATP6V0C, LGMN, AP3D1, AP1M1, AP4S1, LAMP2, ARSB, CTSK, DNASE2, SUMF1, AP4E1 |
| NF-kappa B signaling pathway | 3.1e-05 | 4.6e-04 | 1.94 | 86 | GADD45B, NFKBIA, TNFAIP3, RELB, BCL2, CXCL2, CD14, ICAM1, CARD11, PLCG1, TICAM1, TIRAP, TAB2, PTGS2, TRAF3, TRAF1, BTK, NFKB1, PLAU, BIRC2, BIRC3, MAP3K7, LCK, ATM, CSNK2A1, TAB1, TICAM2, PIAS4, IKBKB, LTA, TAB3, TRAF5, CHUK, IRAK4, TNFSF14, LBP, CARD10 |
| RNA transport | 3.9e-05 | 5.6e-04 | -1.734 | 162 | RAN, EIF4E2, EIF4EBP2, SUMO2, POP4, CLNS1A, GEMIN7, UBE2I, THOC7, SUMO1, EIF1AX, TACC3, POP5, NCBP2, EIF5B, EIF3H, STRAP, SAP18, EIF2B5, SMN2, PYM1, SMN1, EIF3B, ALYREF, RAE1, RBM8A, EIF2S1, EIF3G, PHAX, GEMIN6, ACIN1, SNORD3B-2, EEF1A1, RPP30, POP1, SEC13, EIF4A3, EIF3F, NUP35, EIF4E, SUMO3, EIF3E, NCBP1, TGS1, EIF3I, RANGAP1, FXR2, EIF4G1, EIF2B1, THOC2, NUP107, EIF3J, EIF4G2, NXT2, UPF2, NXF1, NUP37, NUP88, PABPC1, RNPS1, POP7 |
| Epstein-Barr virus infection | 5.2e-05 | 7.0e-04 | -1.676 | 193 | HSPA1B, HSPA8, YWHAB, POLR2K, RAN, POLR2L, YWHAZ, CDK1, PSMD6, POLR2E, YWHAE, HLA-DRB1, YWHAQ, PTMA, POLR2F, PSMC3, CDKN1A, PSMD1, HLA-A, MAP2K3, PSMD12, HSPB1, PSMC2, |

| | | | | | |
|---|---|---|---|---|---|
| | | | | | CCNA2, HDAC1, SEM1, HLA-DQB1, PSMD4, POLR3GL, YWHAH, PSMD7, POLR2G, POLR1D, PSMC5, CDK2, POLR3H, HSPA1A, POLR2C, POLR2J, HDAC2, PSMD8, PSMC4, YWHAG, PSMD14, MAPK11, POLR2I, GTF2E2, POLR2D, MDM2, PSMC6, POLR3K, GTF2E1, ADRM1, HLA-DPA1, PSMC1, CD38, TBPL1, PSMD3, TBK1, CCNA1, PSMD11, TRAF2, PLCG2, GSK3B, MAP2K4, POLR2H, PRKACB, ITGAL, IRAK1, ENTPD8, HLA-DQA2, HLA-DQA1 |
| Glutathione metabolism | 1.1e-04 | 0.001 | -1.945 | 44 | GSTP1, PGD, SMS, GSTO1, GSTM3, TXNDC12, MGST2, IDH1, GSTA3, GPX3, RRM2, GPX4, LAP3, RRM1, SRM, GSTT2B, MGST1, ODC1, GSTA4, MGST3, GSTO2, GPX1, GGT6 |
| Metabolism of xenobiotics by cytochrome P450 | 2.0e-04 | 0.003 | -1.86 | 40 | GSTP1, GSTO1, ADH5, GSTM3, MGST2, CBR3, EPHX1, GSTA3, CYP1B1, CYP2E1, DHDH, CBR1, GSTT2B, MGST1, GSTA4, AKR1C1, MGST3, GSTO2, ALDH3B2 |
| Purine metabolism | 2.4e-04 | 0.003 | -1.624 | 164 | POLR2K, POLR2L, CANT1, HPRT1, POLR2E, NME4, POLR2F, NT5C3B, PKM, NME1, PNPT1, APRT, POLR3GL, NUDT9, POLR2G, PAICS, POLD2, POLE4, PRPS1, POLR1D, PNP, NTPCR, POLR3H, HDDC3, AK2, PGM1, POLR2C, POLR2J, ENTPD2, PDE4B, ATIC, PDE1B, POLR2I, ENTPD6, PDE8B, PRIM2, DCK, POLR2D, RRM2, NUDT2, POLR3K, GUCY2C, RRM1, ALLC, POLA2, IMPDH1, AK1, GUK1, PDE6D, PGM2, AK7, ZNRD1, PDE3B, POLR2H, ENTPD8, PDE6G |
| Systemic lupus erythematosus | 2.9e-04 | 0.003 | -1.846 | 40 | SNRPD3, HLA-DRB1, SNRPD1, HLA-DQB1, C1R, SSB, HLA-DMB, SNRPB, TRIM21, HLA-DPA1, C2, ACTN4, ACTN1, HLA-DQA2, HLA-DQA1 |
| Breast cancer | 3.6e-04 | 0.004 | 1.629 | 146 | GADD45B, JUN, HEY1, GADD45A, JAG1, CSNK1A1, RPS6KB1, EGFR, HEY2, FZD8, AXIN1, PIK3CA, MYC, ERBB2, LRP6, HES5, SHC2, HES1, POLK, RAF1, DLL1, BRCA2, HEYL, GADD45G, NCOA3, NCOA1, BRCA1, DVL1, WNT4, FLT4, PIK3R1, ESR2, WNT9A, TP53, FGF4, NOTCH1, FGF11, KIT, FGF19, SHC3, PIK3CD, DLL4, MTOR, KRAS, FZD1, DVL2, E2F1, FGF2, WNT3, SOS1, FZD7, WNT5B |
| Cell cycle | 4.8e-04 | 0.005 | -1.63 | 124 | PTTG1, YWHAB, CCNB1, PCNA, YWHAZ, RBX1, TTK, CDK1, YWHAE, YWHAQ, CCND1, CCND3, SKP1, SFN, BUB1, CDKN1A, CCNA2, HDAC1, CCNE2, CDKN2C, YWHAH, CCNB2, MAD2L1, CDC20, CDK2, SMC1B, CDC26, MAD2L2, HDAC2, ANAPC13, YWHAG, ANAPC11, CDK7, CDC25A, MDM2, MCM6, TFDP1 |
| Transcriptional misregulation in cancer | 5.0e-04 | 0.005 | 1.586 | 159 | DDIT3, CEBPB, GADD45B, NUPR1, PER2, MITF, CCND2, BCL6, GADD45A, PAX3, CD14, NSD2, CEBPA, CDK9, MYC, LDB1, MMP9, NCOR1, FLT1, POLK, TRAF1, JMJD1C, ETV7, CCNT2, ZEB1, GADD45G, NFKB1, PLAU, SIN3A, KDM6A, BIRC3, |

| | | | | | |
|---|---|---|---|---|---|
| | | | | | FOXO1, NTRK1, KLF3, ZBTB17, ATM, TP53, CDK14, ETV1, BAIAP3, RUNX2 |
| Insulin resistance | 6.9e-04 | 0.006 | 1.714 | 102 | TRIB3, PCK2, NFKBIA, CREB1, GFPT1, RPS6KB1, CREB5, PIK3CA, MAPK9, IRS2, PPARGC1B, PRKAG2, OGT, PRKCZ, STAT3, NFKB1, FOXO1, PIK3R1, SOCS3, PTPRF, PPP1R3B, PDPK1, PRKAA1, MAPK10, SREBF1, IKBKB, PPP1R3C, PIK3CD, PTPN11, MTOR, PTPN1, RPS6KA3, MAPK8 |
| N-Glycan biosynthesis | 6.9e-04 | 0.006 | -1.846 | 47 | DAD1, DPM1, RPN2, MGAT4C, RPN1, DPM3, DPAGT1, ALG6, MGAT1, DPM2, STT3A, DOLK, ALG8, GANAB, STT3B, TUSC3, ALG1, MGAT5B, FUT8, ALG14, B4GALT2, DDOST, MGAT2, MGAT3, DOLPP1, ALG10B, B4GALT3, ALG3, ALG11 |
| Pyrimidine metabolism | 6.1e-04 | 0.006 | -1.704 | 98 | POLR2K, POLR2L, CANT1, POLR2E, DCTPP1, DUT, NME4, POLR2F, NT5C3B, NME1, PNPT1, POLR3GL, POLR2G, POLD2, POLE4, POLR1D, PNP, POLR3H, POLR2C, POLR2J, DTYMK, POLR2I, TYMS, ENTPD6, PRIM2, DCK, POLR2D, RRM2, NUDT2, POLR3K, CTPS2, RRM1, POLA2, UCKL1, DCTD, ZNRD1, POLR2H, DPYS, ENTPD8, DPYD |
| RNA degradation | 5.5e-04 | 0.006 | -1.749 | 74 | LSM3, BTG2, DCP2, WDR61, HSPD1, LSM5, TOB2, ENO1, PNPT1, LSM7, EDC3, LSM8, EXOSC4, LSM4, DDX6, DIS3, LSM2, EXOSC5, EXOSC9, MPHOSPH6, EXOSC10, TOB1, BTG3, PNLDC1, LSM6, ENO3, CNOT2, LSM1, CNOT7, EXOSC1, C1D, PABPC1, PABPC4, BTG4, EXOSC3, PAN3, EXOSC7, CNOT4, DCPS |
| Pathogenic Escherichia coli infection | 8.3e-04 | 0.007 | -1.766 | 51 | TUBB, ACTB, CDC42, ARPC5, RHOA, YWHAZ, TUBB4B, ACTG1, TUBA1C, TUBA1A, ARPC2, YWHAQ, CTNNB1, TUBA1B, TUBB6, ARPC3, KRT18, EZR |
| Chemical carcinogenesis | 9.7e-04 | 0.008 | -1.794 | 45 | GSTP1, SULT1A1, GSTO1, ADH5, NAT2, GSTM3, MGST2, EPHX1, GSTA3, AKR1C2, CYP1B1, CYP2E1, CBR1, GSTT2B, MGST1, GSTA4, SULT1A3, MGST3, GSTO2, ALDH3B2, NAT1 |
| Drug metabolism - cytochrome P450 | 9.9e-04 | 0.008 | -1.814 | 35 | GSTP1, GSTO1, ADH5, GSTM3, MGST2, AOX1, GSTA3, MAOB, CYP2E1, GSTT2B, MGST1, GSTA4, MGST3, GSTO2, ALDH3B2 |
| Pyruvate metabolism | 8.7e-04 | 0.008 | -1.85 | 38 | MDH1, LDHA, LDHB, FH, MDH2, GLO1, PKM, ME3, ALDH7A1, LDHC, ACAT1, ACAT2, ME1, ALDH3A2, ACYP2, ALDH1B1, ACYP1, DLAT, ACSS2, PDHA1, PDHB |
| Terpenoid backbone biosynthesis | 9.5e-04 | 0.008 | -1.891 | 22 | IDI1, ICMT, NUS1, PMVK, FDPS, FNTA, FNTB, ACAT1, ACAT2, PDSS1, MVD |
| Progesterone-mediated oocyte maturation | 0.001 | 0.009 | -1.647 | 88 | HSP90AB1, CCNB1, HSP90AA1, AURKA, GNAI2, CDK1, BUB1, CCNA2, CCNB2, MAD2L1, CDK2, CDC26, SPDYA, MAD2L2, ANAPC13, GNAI3, ANAPC11, MAPK11, CDC25A, GNAI1, ANAPC10, MAPK3, ANAPC5, CCNA1, CDC25B, PDE3B, PRKACB, PRKACA, CDC25C, KIF22 |

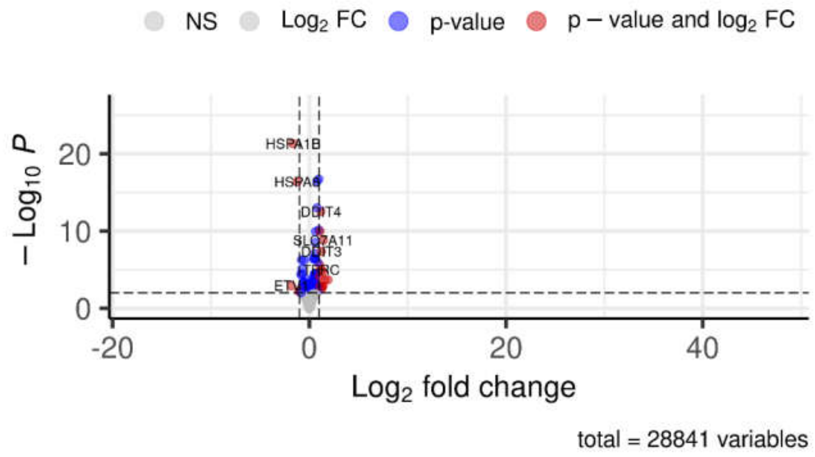


**A**

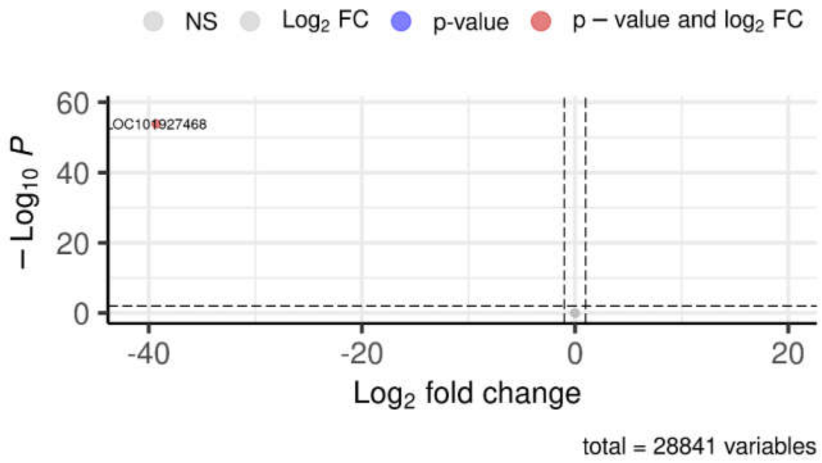


**B**

**Figure S5.** Effect of topotecan and OL9-119 combination on A) WT HEK293A cells treated with Tpc + OL9-119 vs cells treated with Tpc + 0.1% DMSO; B) PARP1-KO HEK293A cells treated with Tpc + OL9-119 vs cells treated with Tpc + 0.1% DMSO. Volcano Plot for differential gene expression under the influence of topotecan and OL9-119.

**Table S4.** Gene set enrichment analysis results of the combination of topotecan and OL9-119 effect on gene expression: pathways significantly associated with differentially expressed genes upon treatment with topotecan and DMSO or topotecan and OL9-119 (WT HEK293A cells treated with 100 nM Tpc + 5 μM OL9-119 vs cells treated with Tpc + 0.1% DMSO).

| Pathway | *p*-Value | *p*-adj | NES | Number | Genes |
|---|---|---|---|---|---|
| Ribosome | 5.3e-10 | 1.6e-07 | -2.23 | 129 | MRPL36, RPS15, RPL36AL, MRPS21, MRPL23, RPL24, MRPS17, RPS27L, RPL35, RPS7, RPL38, RPL18A, RPL34, MRPL16, RPS16, RPL27, RPS26, RPL36, RPS5, RPL28, MRPL27, RPL23, RPL8, RPL18, MRPL33, RPS28, MRPS18C, MRPL20, RPL7A, MRPL28, MRPS7, MRPL24, RPS3, MRPS12, UBA52, RPL41, RPL37A, RPL26, RPL10, RPL35A, RPS17, RPS13, MRPL35, RPL30, RPL7, RPS11, RPS14, RPSA, RPL29, MRPL34, RPL37, RPL21, RPL10A, RPL14, MRPL18, MRPL17, FAU, RPL26L1, MRPL1, RPL11, RPS20, RPS25, RPL17, RPS19, RPS23, MRPL2, MRPL3, RPS29, MRPL21, RPL23A, RPS8, RPL15, RPS2, RPL32, RPS4X, RPS9, RPL39, RPS21, RPS12, RPS10, RPL6, RPL13, RPS27, RPL22, MRPL19, RPL27A, MRPS15 |
| Oxidative phosphorylation | 8.0e-08 | 1.2e-05 | -2.109 | 98 | NDUFA2, NDUFA4, NDUFS5, COX17, NDUFA8, NDUFS3, NDUFA1, NDUFS6, NDUFB7, COX7B, COX8A, COX6B1, ATP6V1B2, ATP6V0E2, SDHD, UQCRH, ATP6AP1, ATP6V0E1, ATP6V0B, NDUFC2, ATP6V1F, NDUFB3, COX4I1, COX7C, UQCR10, COX7A2, NDUFA11, ATP6V0A4, ATP6V0C, NDUFS7, NDUFS8, COX7A2L, NDUFB9, NDUFA3, CYC1, UQCRQ, NDUFB6, NDUFC1, NDUFA6, COX5B, NDUFB4, NDUFB2, ATP6V0D1, NDUFS4, UQCRC1, COX6A1 |
| Huntington's disease | 3.0e-07 | 3.1e-05 | -1.892 | 169 | POLR2L, NDUFA2, NDUFA4, NDUFS5, POLR2E, NDUFA8, NDUFS3, NDUFA1, NDUFS6, POLR2F, NDUFB7, POLR2G, COX7B, COX8A, HDAC1, AP2S1, COX6B1, POLR2C, SDHD, UQCRH, CREB3L2, TBPL1, PPIF, VDAC1, SOD2, NDUFC2, NDUFB3, AP2M1, COX4I1, POLR2B, COX7C, UQCR10, COX7A2, NDUFA11, VDAC3, POLR2K, GPX1, SLC25A5, NDUFS7, NDUFS8, COX7A2L, GRIN2B, NDUFB9, NDUFA3, CYC1, DNAH2, UQCRQ, NDUFB6, DNAH5, NDUFC1, CYCS, DNAI2, POLR2H, NDUFA6, AP2A2, COX5B, SLC25A6, EP300, NDUFB4, NDUFB2, DNAH3, CLTA, NDUFS4, UQCRC1, COX6A1, POLR2J3, DNAH8 |
| Spliceosome | 7.8e-07 | 6.1e-05 | -1.927 | 131 | HSPA1B, HSPA8, SF3B5, HSPA1A, HNRNPM, PPIH, DDX23, CHERP, SNRPG, SRSF3, PRPF3, SNRPD2, SF3B4, ALYREF, LSM7, LSM3, SRSF2, DDX5, LSM2, HNRNPA1, SRSF1, DHX16, U2AF2, RBM8A, SNRPD3, NCBP2, SNRPB, PRPF38B, SF3B3, U2AF1L4, LSM4, HNRNPK, PUF60, ACIN1, ISY1, SNRNP70 |
| Parkinson's disease | 1.2e-06 | 7.7e-05 | -2.012 | 109 | NDUFA2, NDUFA4, NDUFS5, NDUFA8, NDUFS3, NDUFA1, NDUFS6, NDUFB7, COX7B, COX8A, COX6B1, UBE2L3, SDHD, UQCRH, PPIF, UBA1, VDAC1, UBB, ADORA2A, NDUFC2, NDUFB3, COX4I1, COX7C, UQCR10, COX7A2, NDUFA11, VDAC3, PARK7, SLC25A5, NDUFS7, NDUFS8, COX7A2L, NDUFB9, NDUFA3, CYC1, |

| | | | | | |
|---|---|---|---|---|---|
| | | | | | GNAI2, UQCRQ, NDUFB6, NDUFC1, CYCS, LRRK2, NDUFA6, COX5B, SLC25A6, SNCAIP, NDUFB4, NDUFB2, NDUFS4, UQCRC1, COX6A1 |
| Proteasome | 3.9e-06 | 2.0e-04 | -2.164 | 43 | PSME3, PSMA3, PSMD3, PSMB2, PSMB4, POMP, SEM1, PSMD6, PSMB5, PSMB7, PSMC4, PSMC5, PSMD2, PSMC6, PSMC2, PSMB6, PSMB3, PSMD1, PSMD8, PSMF1, PSMC3, PSMA7, PSMB1, PSMA1 |
| Regulation of actin cytoskeleton | 5.7e-05 | 0.003 | -1.641 | 203 | ACTG1, ACTB, MYLK2, CFL1, ARPC5, HRAS, VCL, ITGB7, PDGFC, ACTN4, TMSB4X, PFN1, MYL9, VAV3, BAIAP2, GSN, PIP5K1B, ARPC4, ARPC5L, CDC42, FGF19, PPP1CA, PPP1R12B, FGFR3, MYL12A, GNA12, FGF17, PIK3R3, BUB1B-PAK6, PAK6, VAV1, FGF5, NCKAP1L, SSH2, MYH9, FGF4, DIAPH1, BCAR1, MAPK3, PAK5, PDGFRA, RHOA, SSH3, CHRM1, MAP2K1, MYH10, ACTN1, PPP1R12C, FGFR1, PIP5K1C, FGF13, MYLK, LIMK2, F2, ITGA7, CYFIP1, ITGA11, DIAPH2, PAK4, MAP2K2, FGFR2, FGF20, ARPC2, PIK3R2, MSN, ARPC3, CHRM3, PDGFA, PDGFD, GIT1, VAV2, EZR, ITGA2 |
| Alzheimer's disease | 1.0e-04 | 0.004 | -1.712 | 144 | NDUFA2, NDUFA4, NDUFS5, CALM3, NDUFA8, NDUFS3, NDUFA1, NDUFS6, NDUFB7, COX7B, COX8A, COX6B1, BACE2, SDHD, UQCRH, APOE, RYR3, NDUFC2, NDUFB3, COX4I1, COX7C, UQCR10, COX7A2, NDUFA11, PSENEN, MAPK3, RTN4, BAD, NDUFS7, NDUFS8, COX7A2L, GRIN2B, NDUFB9, NDUFA3, HSD17B10, CYC1, UQCRQ, NDUFB6, NDUFC1, IL1B, CYCS, NDUFA6, COX5B, CAPN1, CACNA1D, NDUFB4, NDUFB2, NDUFS4, UQCRC1, COX6A1, SNCA, ATP2A2, CDK5, MAPT, CACNA1C, UQCR11, GAPDH |
